# Natural products influence bacteriophage infectivity

**DOI:** 10.1039/d5np00014a

**Published:** 2025-08-18

**Authors:** Zhiyu Zang, Joseph P. Gerdt

**Affiliations:** a Department of Chemistry, Indiana University Bloomington IN 47405 USA zzang@iu.edu jpgerdt@iu.edu

## Abstract

Covering: 1942–2025

Bacteriophages (phages) are obligate viruses that infect bacteria. The antibacterial effects of both phages and natural products shape microbial ecosystems and have yielded competing antibiotic strategies. Phages have also intersected many times with natural products research throughout the past century. To discover antiviral leads, natural products were screened for anti-phage activity. To discover new anti-cancer drugs, natural products were screened for the ability to trigger lysis by the λ prophage—indicating DNA damage. Now, the antibiotic resistance crisis motivates the study of natural products that can synergize with phages to improve antibacterial therapies. Beyond applications, these parallel natural “chemical” and “biological” antibacterial factors combine to shape microbial communities across our planet. Here, we provide a comprehensive overview of natural products that modulate phage activities. We discuss their mechanisms of action, and we present opportunities for future research.

## Abstract 2

This review comprehensively reports historical and recent examples of natural products that either prevent bacteriophages from infecting bacteria, promote those infections, or regulate the induction of the lytic cycle of prophages.

## Graphical abstract

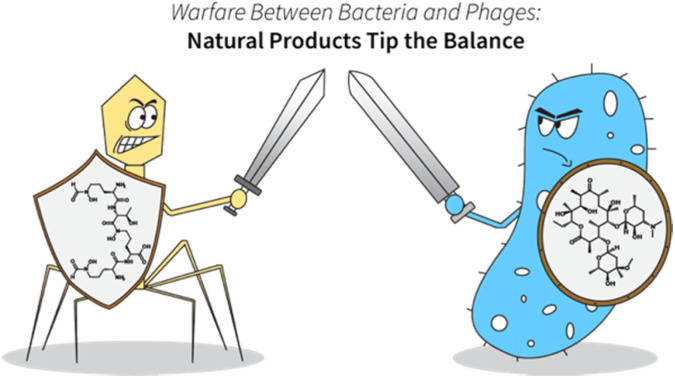

## 1. Introduction

Frederick Twort and Félix d'Hérelle discovered bacteriophages (phages) over a century ago.^1^ Since then, the study of phages has been pivotal for the fields of virology, molecular biology, microbial ecology, and antibacterial therapeutics. As viruses that exclusively infect (and often lyse) bacteria, phages are natural killers of pathogenic bacteria. Shortly after Félix d'Hérelle identified phages, he realized their therapeutic potential and explored the possibility of using phages to treat bacterial infections in both animals and humans.^2^ His early efforts in “phage therapy” pioneered the way bacterial infections are treated today in some parts of the globe—perhaps most notably in the nation of Georgia.^3^ Phage research in the 20th century also led to paradigm-shifting discoveries^4^ including the realization that DNA is nature's hereditary material,^5^ the identification of mRNA as the short-lived intermediate before protein synthesis,^6^ the employment of restriction enzymes in molecular biology,^7^ the development of phage display techniques to identify countless peptide-binding interactions,^8^ and the employment of CRISPR-Cas in genetic engineering.^9^ Furthermore, the recent antibiotic resistance crisis^10^ is reviving global interest in phage therapy.^11^ This renewed excitement in phage research warrants a review of the long history of natural products' influence on phages, as well as a discussion of recent discoveries and avenues for future research.

**Fig. 1:**
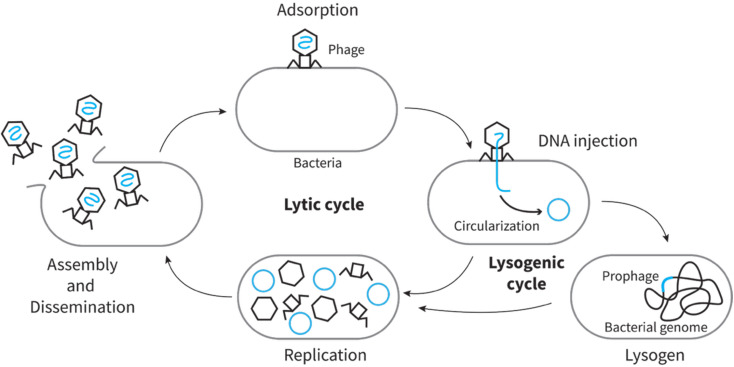
The lytic and lysogenic life cycle of phage.

The two major life cycles found in phages are the lytic cycle and the lysogenic cycle (Fig. 1). Obligately lytic phages only undergo the lytic cycle, kill the host, and release new progeny to the environment (Fig. 1). In contrast, temperate phages can undergo both the lytic and the lysogenic cycles. In the lysogenic cycle, phages integrate their DNA into the bacterial genome and lay dormant within the host as a prophage. When the right conditions arise, the prophage can excise from the host genome and undergo the lytic pathway to infect nearby cells (Fig. 1).

Studies on natural products that influence phage behavior have led to the discovery of anti-viral compounds,^12^ anti-cancer drugs,^13–15^ and phage-antibiotic synergies.^16–19^ Beyond these medical applications, secondary metabolites also shape the natural symbiotic relationships between microbial species by modulating phage activities. On one hand, anti-phage molecules produced by one species may protect a polymicrobial community from phage predation, thus fostering a mutualistic or commensal interaction. On the other hand, metabolites may promote phage infections or induce lysogenic phages into the lytic cycle, which could benefit the metabolite producer by eliminating its competing bacteria.

A revived interest in phages is refocusing some chemists on the interactions of natural products with phages. Phage-promoting natural products may be co-administered with phages to improve phage therapy. In contrast, natural products in the environment or host may inhibit phage therapy, and therefore necessitate alternate strategies. Finally, phage-metabolite synergies may shape microbiome health. These applications justify a deep exploration into the known interactions between natural products and phages (and call for further research to expand the current frontiers).

We note that other recent reviews have discussed small molecules that inhibit phage infections and affect lysis–lysogeny decisions.^20–22^ This review goes beyond to also include small molecules that promote phage infections. As our topic focuses on natural products, synthetic or semisynthetic compounds will be largely excluded from discussion.

This review is organized primarily by the categories of impact on phages. First, we discuss natural products that inhibit phage proliferation. Then, we discuss metabolites that promote phage replication on bacterial hosts. Finally, we discuss natural products that impact the lysis–lysogeny decision of temperate phages. Each category is further divided by the mechanisms by which the natural products carry out their influences on phages. In some cases, the mechanisms are still poorly understood, but we do our best to explain the likely modes of action, given insights from non-phage studies.

## 2. Anti-phage natural products

Dozens of phage-inhibiting natural products have been described over the decades. These discoveries have implications for anti-viral therapy and microbial ecology. Since a molecule that inhibits bacteriophage replication sometimes also inhibits viruses that infect animals,^12,23,24^ anti-phage natural products provide an easy initial screen for the discovery of new anti-viral compounds. Furthermore, in nature, these anti-phage natural products likely shape microbial ecosystems. The evolutionary pressures driving the production of anti-phage natural products are debatable. One hypothesis is that microbial-encoded anti-phage natural products might have evolved as immune mechanisms against phage attacks.^25,26^ Beyond self-immunity, these anti-phage metabolites might also provide “herd immunity” against phage predation for an entire microbial community. However, as discussed below, many (but not all) anti-phage natural products are also antimicrobial. Therefore, it is possible that the production of several anti-phage natural products was primarily driven by their direct influence on microbial competitors—not their anti-phage activity. Regardless of their evolution, anti-phage metabolites have the capacity to shape microbial ecology. They may also diminish the efficacy of phage therapy, warranting attention to the complex chemical environments that can influence phage–bacteria interactions.

**Fig. 2:**
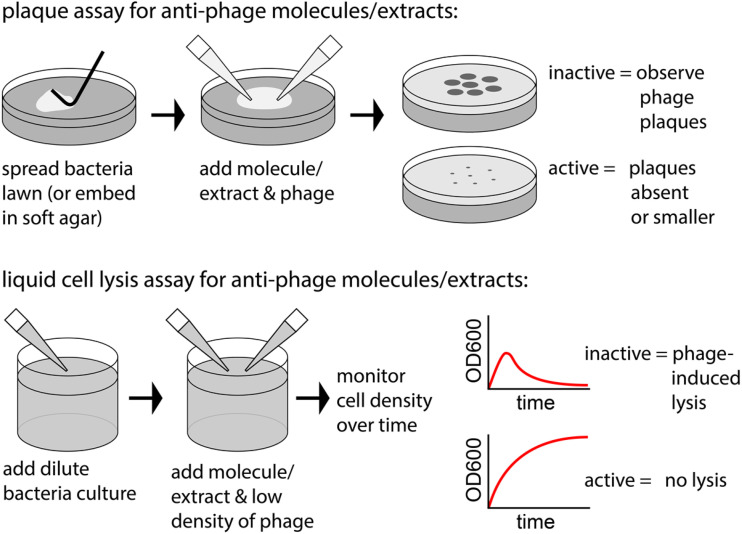
Example experiments to observe anti-phage natural products.

Multiple methods have been employed to assess the anti-phage activity of natural products. We highlight two methods (Fig. 2) that can reveal selective anti-phage activity by molecules that are not antimicrobial (at least at the applied dose). One case monitors the reduction of plaques (areas of phage-induced bacterial lysis on an agar surface). The other case monitors a reduction of phage-induced lysis in liquid culture.

Most anti-phage natural products arrest core phage functions that are also core cellular functions, such as DNA replication, transcription, and protein synthesis. In many cases, anti-phage molecules inhibit phage proliferation more potently than host cell replication. This selectivity may stem from phage-specific molecular targets (*e.g.*, linear DNA and phage-encoded enzymes) being more sensitive to anti-phage molecules. Alternatively, because phages are fast-replicating entities, they may simply be more susceptible to minor perturbations. Apart from inhibiting core functions of genome replication and gene expression, some anti-phage natural products inhibit phage attachment by inducing modifications to the host cell surface. Below, we discuss individual anti-phage natural products, categorized by their likely mechanisms of action.

### 2.1. Interfere with DNA replication and/or transcription

**Fig. 3:**
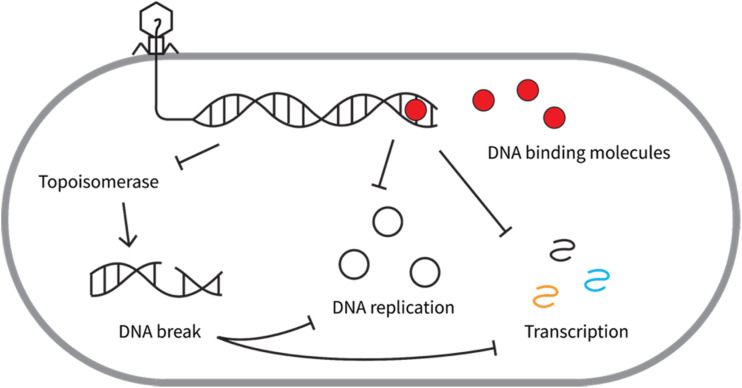
The major mechanisms of action of anti-phage DNA-binding molecules.

DNA-binding molecules are the most commonly observed anti-phage natural products. These molecules antagonize phage reproduction by interfering with phage DNA synthesis and/or transcription (Fig. 3). The binding of these molecules to DNA may stall the movement of DNA/RNA polymerase along DNA or inhibit the coiling and relaxing of DNA by topoisomerases (Fig. 3).^27^ Following are examples of anti-phage natural products that bind DNA.

#### 2.1.1. Anthracyclines

**Fig. 4:**
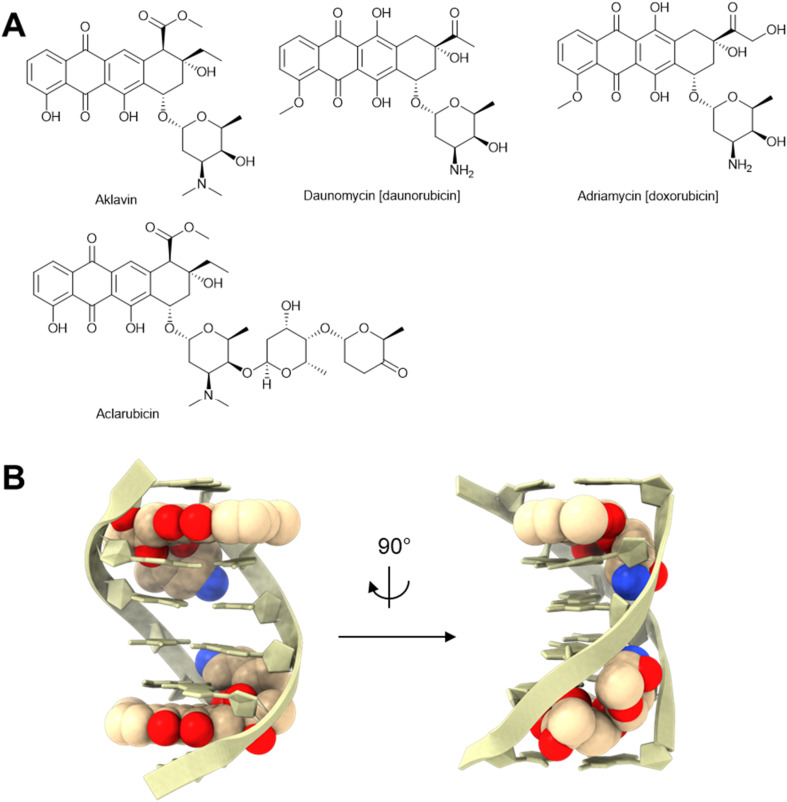
Anthracycline anti-phage molecules. (A) Chemical structures of anthracyclines. (B) Daunorubicin:DNA complex [PDB: 1D10].

Anthracyclines make up a class of antibiotics and chemotherapy drugs. They contain a tetracyclic backbone with an anthraquinone core and a sugar moiety (Fig. 4A). This class of molecules has an extensive research record—primarily due to the antitumor activities of many of its members. The first molecule in this class with reported anti-phage activity was aklavin in 1955, which was purified from Actinomycetia.^28^ It was shown to inhibit phages T2 and T5 forming plaques on *Escherichia coli*, as well as a diverse panel of phages infecting other bacteria.^28^ Later studies in the 1960s and 1970s reported that other molecules belonging to the anthracycline family could specifically inhibit DNA phages but not RNA phages. These selective inhibitors include daunorubicin^29^ (*i.e.*, daunomycin, isolated from *Streptomyces peucetius*^30^), doxorubicin^31^ (*i.e.*, adriamycin, isolated from *Streptomyces peucetius*^32^), and aclarubicin^31^ (*i.e.*, aclacinomycin A, isolated from *Streptomyces galilaeus*^33^).

The specific inhibition of DNA phages suggested a favorable interaction between anthracyclines and DNA. Indeed, anthracyclines interact with DNA by intercalating their planar tetracycline backbone between two alternating C–G base pairs with the amino sugar extended into the minor grove of the DNA double helix, as visualized by X-ray diffraction (Fig. 4B).^34–37^ Because anthracycline binding can stabilize the DNA duplex, these molecules may inhibit phage infection by directly interfering with the action of both DNA polymerase^38^ and RNA polymerase^39^(Fig. 3).

Another possible mechanism to explain the inhibition of anthracyclines on phage DNA replication and transcription is that they can interfere with the function of type II topoisomerases (Fig. 3).^40^ Type II topoisomerase is an important enzyme for DNA replication and transcription during phage infection. It cuts both strands of the DNA helix and reseals them to manage DNA tangles and supercoils.^41^ For example, T-even phages encode their own type II topoisomerase, which is required to relax potential DNA supercoils or to resolve DNA knots of the rapidly replicating genome.^42,43^ With anthracycline intercalated into DNA, a stable ternary complex forms among anthracycline, DNA, and topoisomerase, which prevents the ligation of double-stranded DNA breaks (DSBs) by the topoisomerase.^44^ Anthracyclines might also induce DSBs through a radical mechanism due to the presence of quinone moiety.^45^ These irreversible DSBs inhibit DNA replication and transcription (Fig. 3),^46^ presumably hampering the phage infection process.

With several potential mechanisms, there is still uncertainty about how anthracyclines inhibit phage infection. As for lysogenic phages, a recent study showed that anthracyclines did not prevent the λ phage genome from entering the cell but significantly reduced the ability of the phage genome to integrate into the bacterial chromosome as a lysogen.^25^ The exact mechanism by which anthracyclines inhibit lysogen formation is still unclear. Surprisingly, anthracyclines did not inhibit λ phage replication after induction of the temperature-sensitive λ prophage.^25^ This discovery complicates the phage inhibition mechanism of anthracyclines, because they do not seem to universally inhibit DNA replication and transcription of all phages. Future investigations on the interaction between anthracyclines and phages may unravel the mechanism behind their selective anti-phage activity.

Although anthracyclines are also anti-bacterial, the phage genome is suspected to be more susceptible to DNA intercalators compared to the bacterial genome, partly because phage DNA is linear, non-supercoiled, and unprotected by DNA-binding proteins when it is injected into the bacterial host.^25^ Therefore, at low doses, anthracyclines can selectively inhibit phages more than their host bacteria.^25,29,31^

#### 2.1.2. Neopluramycin

**Fig. 5:**
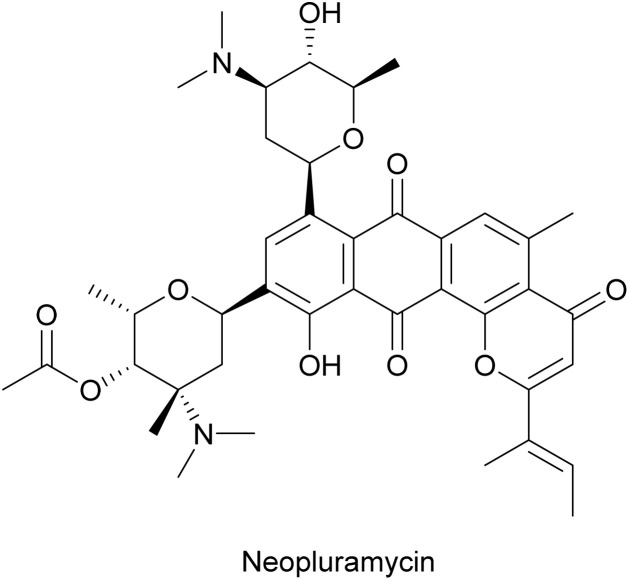
Chemical structure of neopluramycin.

Neopluramycin was first isolated from *Streptomyces pluricolorescens* in 1970 and exhibited antibiotic and anticancer activities.^47^ Shortly after its discovery, neopluramycin was also found to inhibit the production of T4 phage particles in *E. coli*.^48^ The phage inhibition activity was due to interference with phage transcription as measured both *in vitro* and in infected cells.^48^ Neopluramycin has a tetracyclic backbone similar to anthracyclines but with a pyran ring fused to the anthraquinone chromophore (Fig. 5). The planar backbone presumably allows neopluramycin to intercalate between two adjacent base pairs with the two amino sugars residing in the minor groove as inferred by NMR studies on its analogue, hedamycin.^49–51^ As with the anthracyclines, the antiphage effect of neopluramcyin probably results from its DNA-intercalating properties, which may not only interfere with phage transcription but also with DNA synthesis (Fig. 3).

#### 2.1.3. Gilvocarcin family of *C*-aryl glycosides

**Fig. 6:**
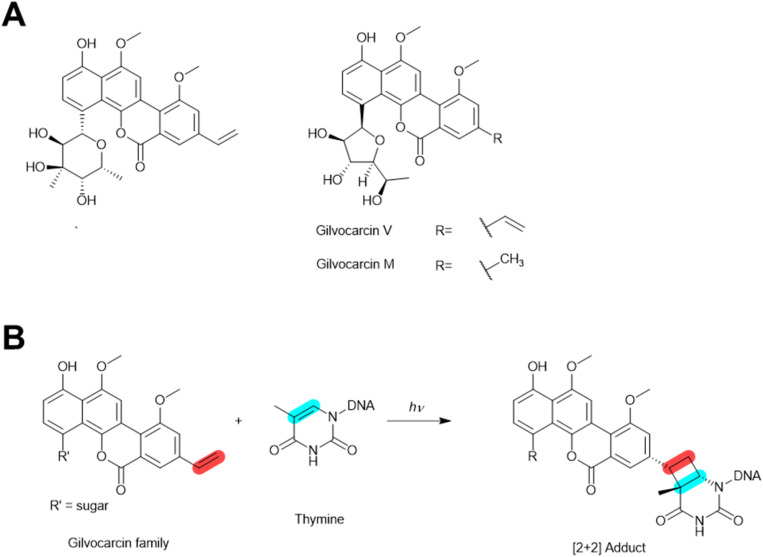
Gilvocarcin anti-phage molecules. (A) Chemical structures of gilvocarcins. (B) Photo-activated DNA alkylation by gilvocarcins.

The gilvocarcin *C*-aryl glycosides (also referred to as benzo[*d*]naphtho[1,2-*b*]pyran-6-one *C*-glycosides) are known for their excellent antitumor activity and remarkably low toxicity.^52^ This family of natural products contains a tetracyclic naphthocoumarin backbone and a vinyl substituent at the C8 position, with various sugars attached to the C4 position of the aromatic backbone *via* a C–C bond (Fig. 6A). Chrysomycin A was the first molecule discovered within this family. It was isolated from a *Streptomyces* bacterium in 1954.^53^ Chrysomycin A inhibits plaque formation by a variety of phages, including coliphages T1 and T2, *Bacillus* phages, *Staphylococcus* phages, and *Enterococcus* phages.^53^ Another member in this family, gilvocarcin V (toromycin), was also shown to inhibit phage infection by phi170, T1, T3, and T5 phages in 1979. The same study reported inhibition of DNA viruses of animals like the vaccinia virus and the herpes simplex virus, but not RNA viruses like the Newcastle disease virus.^23^ Later it was shown that gilvocarcin V can bind to single-stranded DNA of coliphage M13 *in vitro*.^54^

The mechanism of inhibition of the gilvocarcin family against phages is still unclear. We speculate that they hinder phage replication by inhibiting phage DNA synthesis through photo-activated DNA alkylation (Fig. 6B). The alkylation relies on visible light or low energy UV radiation, which initiate a [2 + 2] photocycloaddition between the gilvocarcin vinyl group and thymine residues of DNA (Fig. 6B),^55^ thereby inhibiting DNA synthesis and causing DNA damage.^56,57^ Moreover, gilvocarcins might interfere with phage DNA synthesis and transcription through the inhibition of topoisomerases (Fig. 3), as chrysomycin A has been shown to inhibit the activity of both type I and type II topoisomerase, presumably through binding to DNA and/or blocking the topoisomerase active sites.^58,59^

Beyond the phage inhibition activity of gilvocarcin family molecules, they can also trigger prophage induction by causing extensive DNA damage in host cells,^60^ which is discussed later in the prophage induction section.

#### 2.1.4. Nybomycin

**Fig. 7:**
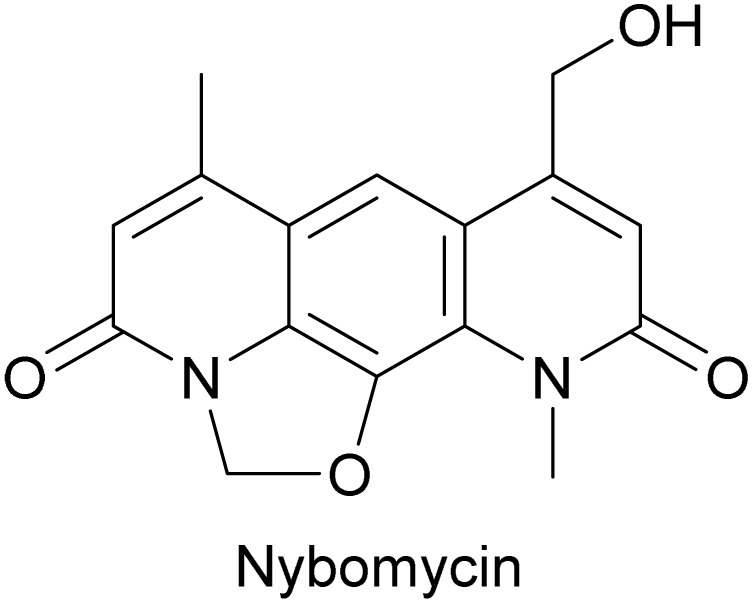
Chemical structure of nybomycin.

Nybomycin is a pyridoquinolinedione-based antibiotic first collected from an Actinomycetia isolate in 1955 (Fig. 7).^61^ The authors tested its ability to restrict plaque formation by a panel of phages and found 33 out of 61 phages were inhibited by nybomycin, including phages of *E. coli*, *Staphylococcus*, *Bacillus*, and *Streptomyces*.^61^

Although the exact mechanism of phage inhibition by nybomycin is still unclear, it likely acts *via* DNA intercalation and/or inhibition of type II topoisomerase. Molecular docking shows that its planar pyridoquinoline structure enables nybomycin to partially intercalate into a DNA double helix.^62^ Additionally, nybomycin can inhibit type II topoisomerase *in vitro* (presumably by stabilizing the nicked DNA-topoisomerase complex), which disrupts DNA supercoiling and relaxation.^62^ Therefore, it is likely that nybomycin's DNA intercalation and/or topoisomerase inhibition prevents phage DNA replication and/or transcription as discussed above (Fig. 3).

#### 2.1.5. Oligopyrrole/polyamide

**Fig. 8:**
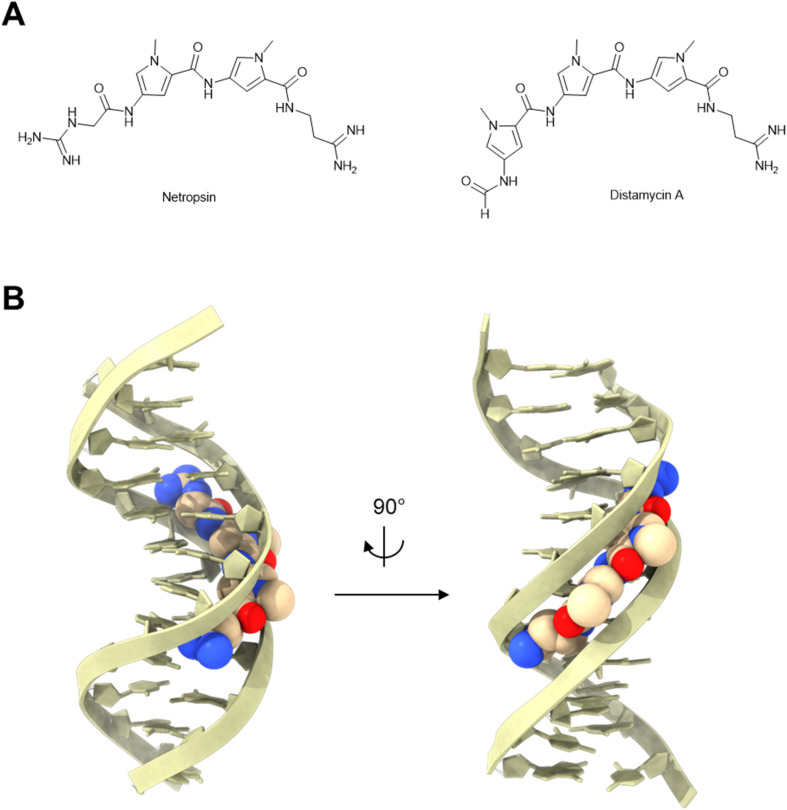
Oligopyrrole anti-phage molecules. (A) Chemical structures of oligopyrroles. (B) Netropsin:DNA complex [PDB: 6BNA].

Netropsin (*i.e.*, T-1384, congocidine, or sinanomycin) and distamycin A are two naturally occurring amide-linked oligopyrrole antibiotics (Fig. 8A) isolated from *Streptomyces netropsis*^63^ and *Streptomyces distallicus*,^64^ respectively in the 1950s. Distamycin A was reported to inhibit phage T1 (ref. 65) and T2 (ref. 66) infection in *E. coli* as evidenced by impeded plaque formation and protection from phage-induced host culture lysis.^65^ Although distamycin A can arrest bacteria growth, it selectively inhibited phage replication at low concentrations that do not inhibit growth of the host bacteria.^65^ Notably, the anti-phage activity of distamycin A also inspired its subsequent investigation as an inhibitor of animal viruses.^12^ Netropsin has not been tested against phages. However, it inhibits the proliferation of several animal viruses, such as vaccinia,^67^ influenza,^68^ and Shope fibroma.^69^ Therefore, it is likely to inhibit phages, as well.

The anti-phage effect of oligopyrrole antibiotics is presumably due to their specific binding within the minor groove of the DNA double helix (Fig. 8B).^70–72^ The binding of oligopyrroles to DNA can interfere with phage DNA replication and transcription in multiple ways. First, distamycin A has been shown to directly inhibit both DNA^73^ and RNA^74^ synthesis *in vitro*.^75^ Phage transcription is preferentially inhibited by distamycin A, compared to bacterial transcription. Namely, a direct *in vitro* comparison revealed that distamycin A inhibited phage T3 RNA polymerase more strongly than an *E. coli* RNA polymerase.^76^ This discovery is in agreement with the selectivity of distamycin A to inhibit phage replication at concentrations that do not arrest bacterial growth.^65^ Second, the binding of distamycin to the DNA minor groove can prevent the catalytic activity of both type I^77^ and type II^78^ topoisomerases by blocking the enzyme binding sites. As discussed earlier, topoisomerase inhibition can indirectly interfere with DNA replication and transcription, thus blocking rapid phage replication (Fig. 3).

#### 2.1.6. Actinomycins

**Fig. 9:**
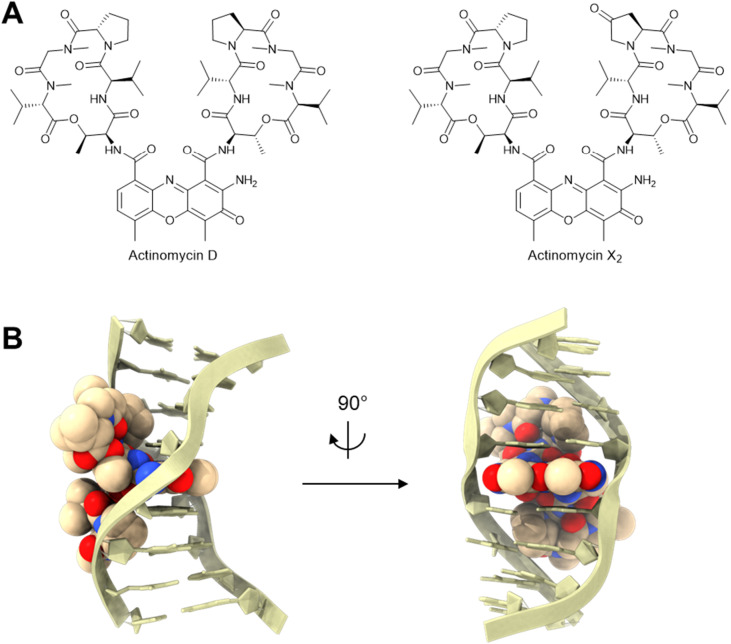
Actinomycin anti-phage molecules. (A) Chemical structures of actinomycins. (B) Actinomycin D:DNA complex [PDB: 2D55].

Actinomycins are a class of chromopeptides with potent cytotoxicity and antimicrobial activity.^79^ Actinomycins feature a phenoxazinone chromophore tethered to two cyclic pentadepsipeptides *via* amide bonds (Fig. 9A). It was first reported in 1961 that a mixture of actinomycins (referred to as actinomycin S) isolated from *Streptomyces flaveolus* 1048A^80^ inhibited multiplication of phage T2 on *E. coli* while not interfering with host growth.^81^ A following study showed that the two major components in actinomycin S were actinomycin D and actinomycin X_2_ (Fig. 9A).^82^ Later, it was shown that actinomycin D can inhibit infections by other phages as well, including *E. coli* phage T4,^83^ and *Bacillus subtilis* phages PBS1 and SP10.^84^ Around the same time, actinomycin D was also shown to inhibit the single-stranded RNA virus that causes foot-and-mouth disease in animals.^85^

The planar structure of tricyclic phenoxazinone in actinomycins allows them to intercalate selectively between alternating G–C base pairs, while the two cyclic pentadepsipeptides can bind in the minor groove of duplex DNA (Fig. 9B).^86,87^ This DNA binding inhibits RNA synthesis both *in vitro*^88^ and in bacteria,^89^ due to the inhibition of RNA elongation by RNA polymerase.^90^ However, it has been shown that actinomycins can inhibit *E. coli* phage reproduction without significantly affecting RNA, DNA, or protein synthesis in the infected cells.^83,91^ In this case, the anti-phage effect may be due to inhibition of DNA packaging into the phage capsid.^83^ Furthermore, the large molecular weight of actinomycins (>1200 Da) restricts their permeability into bacterial cells, especially in gram-negative bacteria.^92^ Therefore, some have suggested that actinomycins perform their anti-phage actions outside the bacterial cells. For example, actinomycins may interfere with the injection of phage DNA by intercalating the ejected DNA at the cell wall. In support of this hypothesis, incubation with actinomycins increased phage DNA injection into the media instead of into cells.^84,91^ Nevertheless, the insensitivity of some DNA phages to actinomycins^65^ suggests that this DNA injection inhibition model is not universal. In one case, synergy between actinomycins and phages was even observed, where the M13 phage infection made *E. coli* more susceptible to actinomycin D.^93^ Further efforts are needed to reveal the true impact of each of these possible mechanisms of inhibition.

#### 2.1.7. Pyrrolobenzodiazepine

**Fig. 10:**
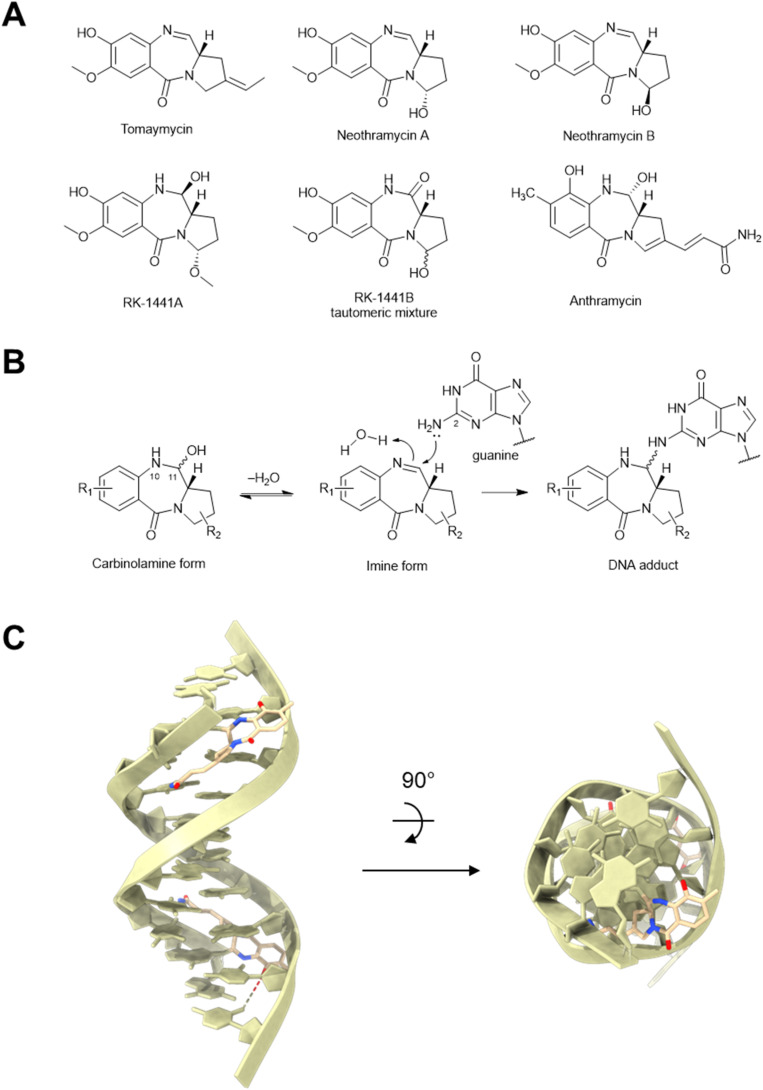
Anthramycin anti-phage molecules. (A) Chemical structures of anthramycins. (B) Anthramycin DNA alkylation mechanism (C) Anthramycin-DNA adduct [PDB: 274D].

Pyrrolobenzodiazepines are naturally occurring antibiotics and antitumor drugs produced by Actinomycetia bacteria.^94^ Pyrrolobenzodiazepines are characterized by tricyclic ring systems consisting of an anthranilate, a 1,4-diazepine, and a hydropyrrole (Fig. 10A). In 1972, tomaymycin isolated from *Streptomyces achromogenes*, was reported to inhibit plaque formation from multiple *E. coli* and *B. subtilis* phages.^95^ Following tomaymycin, other members in this family were also shown to inhibit phage infection in both *Streptomyces griseus* and *E. coli*,^96^ such as neothramycin^97^ (a mixture of stereoisomers A and B, which interconvert in aqueous solution), RK-1441A,^96^ and RK-1441B.^96^

As with many of the previously mentioned molecules, pyrrolobenzodiazepines likely inhibit phage replication by interrupting DNA synthesis^98^ and transcription (Fig. 3).^99^ The imine group at N-10 and C-11 in pyrrolobenzodiazepines can covalently bind the NH_2_-2 group of guanine in DNA (Fig. 10B and C).^100^ The carbinolamine form (*e.g.* anthramycin and RK-1441A) can undergo water elimination first^101^ to form an imine intermediate and then alkylate DNAs. The S configuration at C-11a confers pyrrolobenzodiazepines a right-handed twist from the anthranilate to the hydropyrrole ring, allowing them to fit perfectly in the minor groove of the DNA double helix (Fig. 10C).^100^ It is surprising that RK-1441B was also active against phage infections, as the stable amide between N-10 and C-11 is not reactive *in vitro* with purified phage DNAs.^96^ It was proposed that the amide might be converted into the active carbinolamine or imine form in the host cells,^96^ which would allow RK-1441B to alkylate DNA as well.

#### 2.1.8. Lanthipeptide

**Fig. 11:**
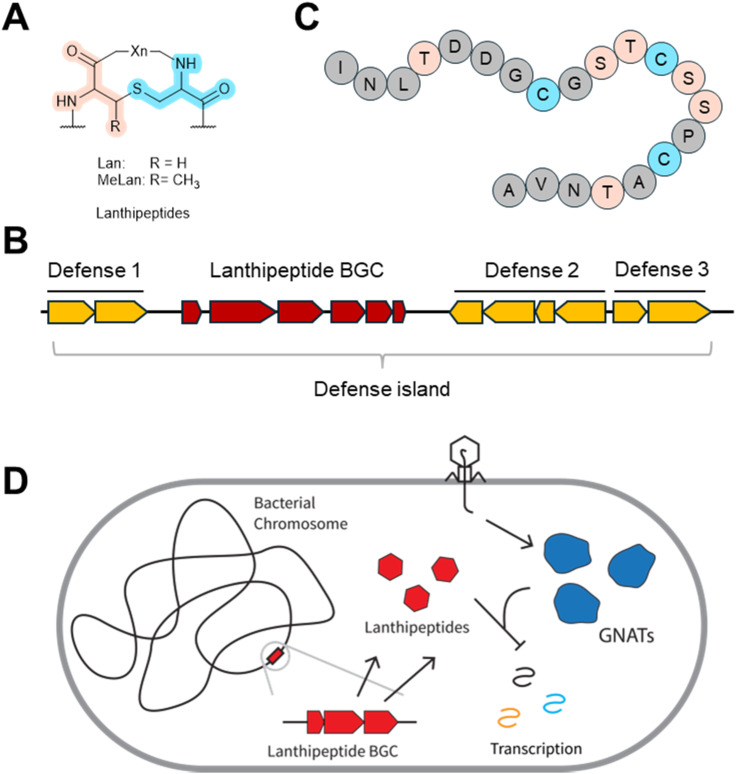
Lanthipeptide anti-phage molecules. (A) Chemical structure of lanthipeptides characterized by the presence of lanthionine (Lan) and methyllanthionine (MeLan). (B) Lanthipeptide BGCs tend to cluster with other anti-phage defense systems within defense islands on bacterial genomes. (C) The core sequence of a representative anti-phage lanthipeptide. (D) Proposed mechanism of action of anti-phage lanthipeptides.

Lanthipeptides are one of largest and most diverse families of ribosomally synthesized and post-translationally modified peptides (RiPPs).^102^ Lanthipeptides are named after the presence of their characteristic β-thioether linked bis-amino acid structures, lanthionine (Lan) and methyllanthionine (MeLan) (Fig. 11A), which are formed by cysteine residues crosslinking with dehydrated serine or threonine residues, respectively.^103^ Lanthipeptides are notable for their antimicrobial,^104^ anti-cancer,^105^ and anti-animal virus^105^ properties. Recently the first anti-phage lanthipeptide was reported.^26^ Through bioinformatics analysis of the genomes from Actinomycetota, the authors discovered that lanthipeptide biosynthetic gene clusters (BGCs) reside near other anti-phage systems within defense islands (Fig. 11B) at a frequency of 8.8%.^26^ This observation suggested that these lanthipeptides serve as anti-phage defenses for the host.^106,107^ Indeed, upon induced native expression or heterologous expression, lanthipeptide BGCs provided robust protection against phage infections in *Streptomyces* (Fig. 11C, predicted core peptides are shown, intramolecular β-thioether linkages are yet uncharacterized).^26^ The lanthipeptide inhibited phage transcription, particularly the late genes.^26^ By comparing the genomes of wild type phages with their lanthipeptide-immune escape mutants, the authors discovered that each of the escaping phages carried a mutant Gcn5-related *N*-acetyltransferase (GNAT). Phage-encoded GNATs are important for shifting between early and late gene expression.^108^ Therefore, the lanthipeptide might inhibit transcription through a yet unknown GNAT-dependent mechanism (Fig. 11D).^26^ It is noteworthy that only intracellular lanthipeptides were found active against phage infections in *Streptomyces* so far.^26^ Therefore, further investigation is required to determine if secreted lanthipeptides from one bacteria can inhibit phage infection in another bacteria. If not, these peptides may only be retained within the producing cell for its own defense.

Perhaps more than any other anti-phage natural product, the main purpose of these lanthipeptides appears to be anti-phage defense. Their biosynthetic genes are located in “phage defense islands”, and the lanthipeptides do not exhibit obvious antibiotic activity. This case contrasts with the previously discussed anti-phage natural products that are also antibiotics. The dual anti-phage/antibiotic activity of the other molecules adds to an existing debate about the evolved roles of naturally occurring antibiotics to benefit the producing organism. As others have noted, antibiotics may mediate microbial competition by killing competitors, or they may serve as signal molecules to regulate transcriptional profiles.^109^ Here we note the third possibility: antibiotics with anti-phage activities might have evolved as immune mechanisms against phage infections. Despite this debate, the case of anti-phage intracellular lanthipeptides appears fairly clear—they likely evolved for defense against phages.

### 2.2. Interfere with peptide synthesis

**Fig. 12:**
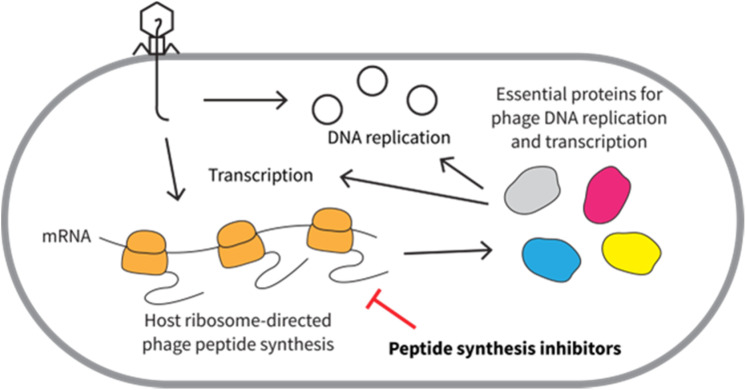
Hypothetical anti-phage mechanisms of peptide synthesis inhibitors.

Peptide synthesis inhibitors comprise another large group of anti-phage natural products. These molecules target the bacterial ribosome. Since phage protein synthesis exclusively relies on host ribosomes,^110^ the inhibitors of host ribosomes also interfere with the synthesis of phage-encoded peptides, thereby reducing phage reproduction (Fig. 12).

#### 2.2.1. Aminoglycosides

**Fig. 13:**
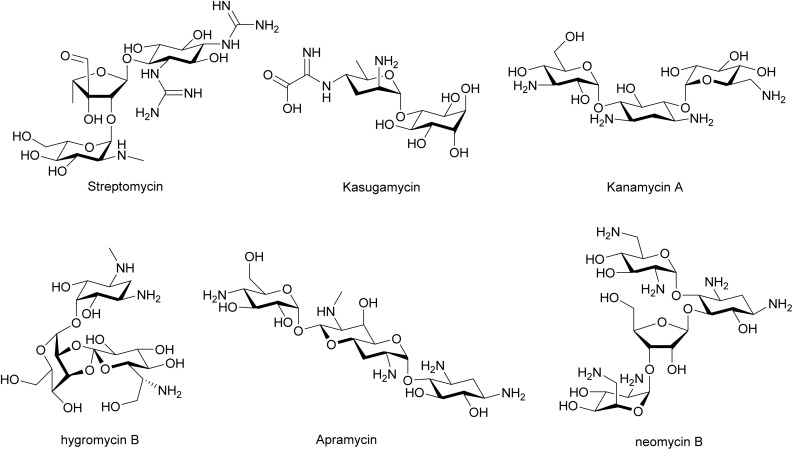
Chemical structures of anti-phage aminoglycosides.

Aminoglycosides were among the first antibiotics to be introduced for clinical treatment of bacterial infections, and they remain one of the major classes of antibiotics in use today.^111,112^ Aminoglycosides feature a set of sugars, amino sugars, and pseudo sugars (*e.g.*, cyclitols and aminocyclitols) that are connected *via* glycosidic linkages (Fig. 13). Streptomycin was the first reported aminoglycoside, isolated from *S. griseus* by Selman Abraham Waksman and colleagues in 1944.^113^ Soon after its discovery, streptomycin was reported to suppress plaque formation and phage multiplication in both *E. coli* and *Staphylococcus aureus* in 1945.^114^ In the following decades, the anti-phage effect of streptomycin was demonstrated against many other phages.^115–120^ Other aminoglycosides beyond streptomycin, such as kasugamycin,^121^ kanamycin A,^120,122,123^ hygromycin B,^120,123^ apramycin,^123^ and neomycin B,^122,124^ have also proven active against phage infection.

**Fig. 14:**
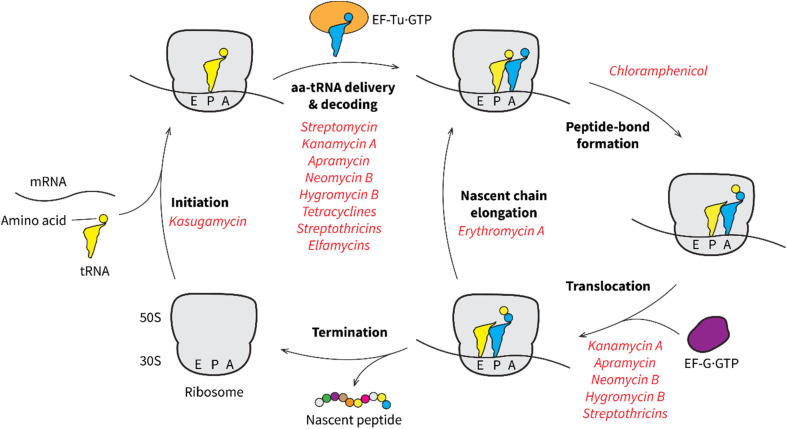
Scheme of peptide synthesis and ribosome recycling. Peptide synthesis is initiated by the formation of a complex between the 70S ribosome (including a small 30S subunit and a large 50S subunit), mRNA, and the initiator tRNA at the P-site. The elongation cycle involves four steps. First, an aminoacylated tRNA (aa-tRNA) is delivered to the A-site with the help of elongation factor Tu (EF-Tu). Upon delivery, the ribosome ensures the correct pairing between the tRNA anti-codon and the mRNA codon (decoding). Next, the amino acid (or peptide in the elongation process) from the P-site tRNA is transferred to the aa-tRNA at the A-site, and a peptide bond is formed. The ribosome-tRNA complex then translocates to the next codon on the mRNA with the help of elongation factor G (EF-G), so that the next aa-tRNA can be delivered to the A-site. In the elongation process, the nascent peptide chain passes through the exit tunnel in the 50S subunit. The elongation cycle terminates when a stop codon is encountered and the nascent peptide chain is released from the ribosome. Steps that are inhibited by natural products are indicated.

The mechanism of antiphage activity from aminoglycosides can be related to their specific interaction with the 30S or 50S subunits of the bacterial ribosome, thus inhibiting distinct steps in protein translation, such as tRNA delivery and selection, ribosome translocation, and ribosome recycling (Fig. 14).^125–132^ As host ribosomes are essential for phage protein synthesis,^110^ malfunction of host ribosomes should interfere with production of phage proteins. It is plausible that the higher demands of phages for fast replication makes them even more susceptible than their hosts to subtle ribosome inhibition by low concentrations of aminoglycosides, affording anti-phage functions at sub-inhibitory doses.

A recent study proposed another anti-phage mechanism of aminoglycosides by directly inhibiting phage DNA replication and transcription.^123^ The authors discovered that apramycin treatment led to a significant reduction in phage DNA replication and transcription.^123^ Since *in vitro* studies have shown that aminoglycosides can cause condensation of purified phage DNA,^133^ the authors suggested that the impaired phage DNA replication and transcription was due to direct binding of aminoglycosides to the unprotected phage DNA following the injection.^123^ Alternatively, the decreased phage DNA and RNA synthesis might result from the impaired function of host ribosomes under aminoglycoside treatment. Phage-encoded proteins are often vital for efficient phage DNA and RNA synthesis. They arrest host gene expression, redirect host DNA and RNA polymerases to phage genomes, assist the initiation of DNA replication, and regulate transcription kinetics.^134–138^ Since phages rely on the host ribosomes for their protein synthesis,^110^ the inhibitory actions of aminoglycosides on the host ribosomes may be the root cause of the observed decrease in phage DNA replication and transcription (Fig. 12).

#### 2.2.2. Tetracyclines

**Fig. 15:**
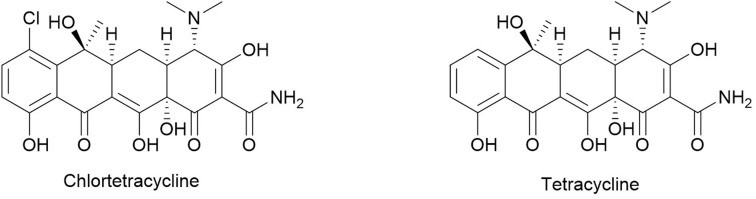
Chemical structures of tetracyclines.

Tetracyclines are a class of broad-spectrum antibiotics characterized by a rigid fused tetracyclic core with a variety of functional groups attached.^139^ In 1948, the first molecule in this class was isolated from *Streptomyces aureofaciens*, named aureomycin (*i.e.* chlortetracycline, Fig. 15).^140^ A few years later, chlortetracycline was shown to inhibit phage T3 infection in *E. coli* by slowing down phage reproduction and reducing its burst size (the number of new phages produced by each infected cell).^24^ In addition, the authors showed that chlortetracycline inhibited phage adsorption onto the host bacteria.^24^ As with many other anti-phage metabolites, chlortetracycline also inhibited an animal virus.^141^ The non-chlorinated analog, tetracycline (Fig. 15), was also reported to inhibit the T3 phage recently.^122^ In that report, tetracycline did not inhibit phage adsorption. The different effects of tetracyclines on phage adsorption is intriguing because chlortetracycline and tetracycline only differ by a chloro group. Further investigation may be warranted to elucidate the importance of the chloro group in antagonizing phage adsorption. Nonetheless, the consistent inhibitory effect of tetracycline on phage reproduction is probably due to the impaired ribosomal function as tRNA delivery is inhibited.^142,143^

The anti-phage effect of tetracycline also intersects with a bacterial defense system (CRISPR-Cas) and a phage-encoded anti-CRISPR (Acr) system. In one case, bacteriostatic antibiotics like tetracycline, chloramphenicol, and erythromycin, promoted CRISPR immunity in a *P. aeruginosa* population by slowing the phage maturation process, thus allowing more time for spacer acquisition.^144^ In another case, the infection of CRISPR-containing *P. aeruginosa* by Acr-encoding phages was also inhibited by tetracycline and the other translation inhibitors chloramphenicol and erythromycin.^145^ These translation inhibitors delayed the production of phage-encoded “immunosuppressing” Acr proteins, thereby allowing the CRISPR immune system to inhibit phage reproduction.^146^

#### 2.2.3. Chloramphenicol

**Fig. 16:**
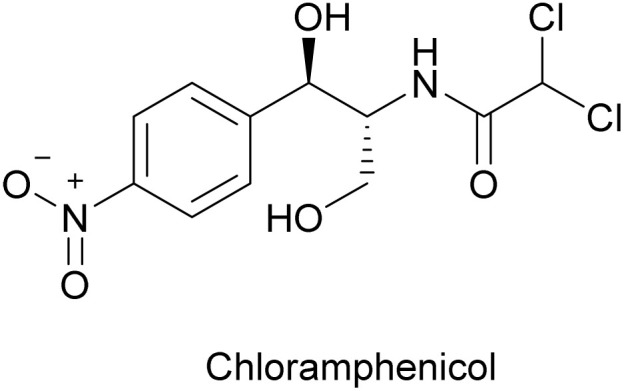
Chemical structure of chloramphenicol.

Chloramphenicol (chloromycetin) is a small molecular weight antibiotic (Fig. 16) originally isolated from *Streptomyces venezuelae* in 1947.^147^ The anti-phage effect of chloramphenicol was first reported in 1954 on *E. coli* phage T1, where bacteriostatic concentrations of chloramphenicol completely arrested phage multiplication in the host cells.^148^ Subsequent studies showed that chloramphenicol is active against a wide panel of coliphages^149–151^ and *Streptococcus* phages.^152^ Chloramphenicol treatment was shown to not affect phage adsorption and DNA penetration^150^ but to inhibit phage protein synthesis.^153^ The protein synthesis inhibition was reversible (*i.e.*, it was relieved after removing chloramphenicol from phage-infected cells).^153^ In some cases, chloramphenicol also inhibited phage DNA synthesis, which is presumably due to the indirect effect of peptide elongation inhibition,^125,150,154–156^

In addition to the direct inhibitory action of chloramphenicol on phage protein synthesis, an earlier study reported that chloramphenicol-resistant bacteria also exhibited resistance to phages.^157^ Chloramphenicol-resistant *Streptococcus* mutants, which evolved during chloramphenicol treatment, became resistant to phage infections. The mechanism of dual resistance to the antibiotic and phages was unclear. The authors found no evidence that the bacterial cell wall was modified in the mutants. In fact, the phages were able to adsorb and inject their genome into the mutants as well as they could into wild-type bacteria. The chloramphenicol-resistant ribosomes may somehow be immune to hijacking by phages.

Chloramphenicol treatments have also been shown to encourage the temperate coliphage P1 to enter its lysogenic phase, while the detailed mechanism is still unclear.^158^

#### 2.2.4. Erythromycin A

**Fig. 17:**
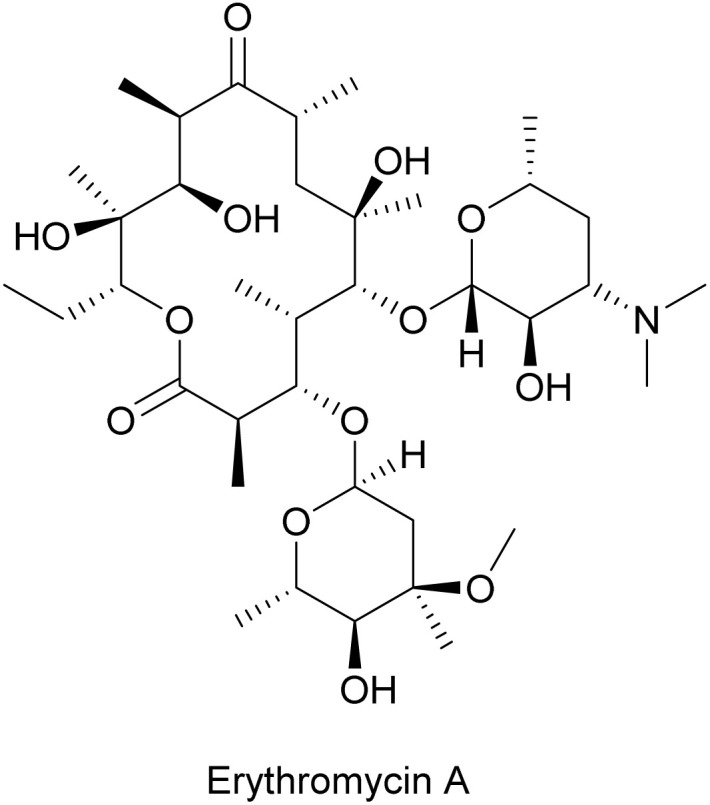
Chemical structure of erythromycin A.

Erythromycin A is a macrolide antibiotic, comprising a 14-membered macrocyclic lactone with two sugar moieties attached (Fig. 17).^159^ Erythromycin A was first isolated from *Saccharopolyspora erythraea* in 1952,^160^ and later was shown to inhibit SPO1 phage multiplication in *B. subtilis* due to impeded phage protein synthesis.^161^ Erythromycin A interacts with host ribosomes and only allows the synthesis of short peptides with 6–8 amino acids before translation aborts (Fig. 14).^125,154,155^ As discussed above, the inhibitory action of erythromycin A on host ribosomes is likely the reason why phage protein synthesis is also inhibited during infection, as phage protein synthesis solely relies on host ribosomes. Slight inhibition of phage DNA synthesis was also observed upon erythromycin A treatment, possibly as a result of hampered synthesis of phage-encoded DNA replication machinery.^161^

#### 2.2.5. Streptothricin

**Fig. 18:**
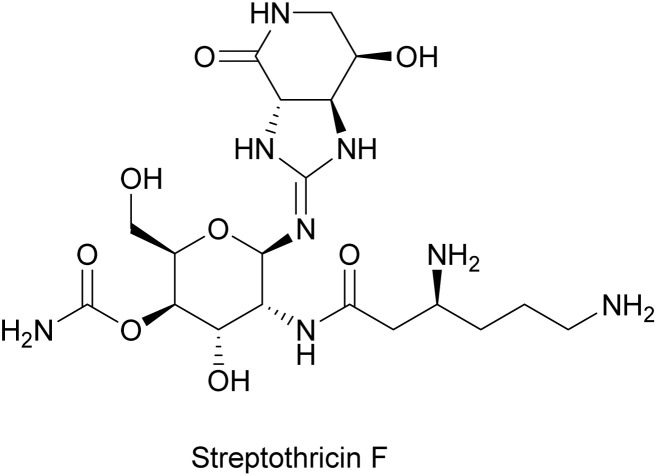
Chemical structure of streptothricin F.

Streptothricins were among the first antibiotics discovered from soil Actinomycetota.^162^ Streptothricin F is the major component of an antibiotic mixture that was first isolated from *Streptomyces lavendulae* in 1942,^163^ which features a streptolidine lactam ring, a glucosamine sugar, and a β-lysine (Fig. 18). Soon after its discovery, the anti-phage activity of streptothricin F was reported against two *E. coli* phages in 1945, where both plaque formation and phage multiplication were inhibited.^114^ Subsequent work revealed inhibition of influenza virus, as well.^164^ Streptothricin F is a protein synthesis inhibitor^165^ that results in miscoding during peptide elongation^166^ and impeded ribosomal translocation^167^ (Fig. 14). The phage inhibition effect from streptothricin F is likely due to its inhibitory action on host ribosomes, thus interfering with the expression of essential phage proteins as discussed above.

#### 2.2.6. Elfamycins

**Fig. 19:**
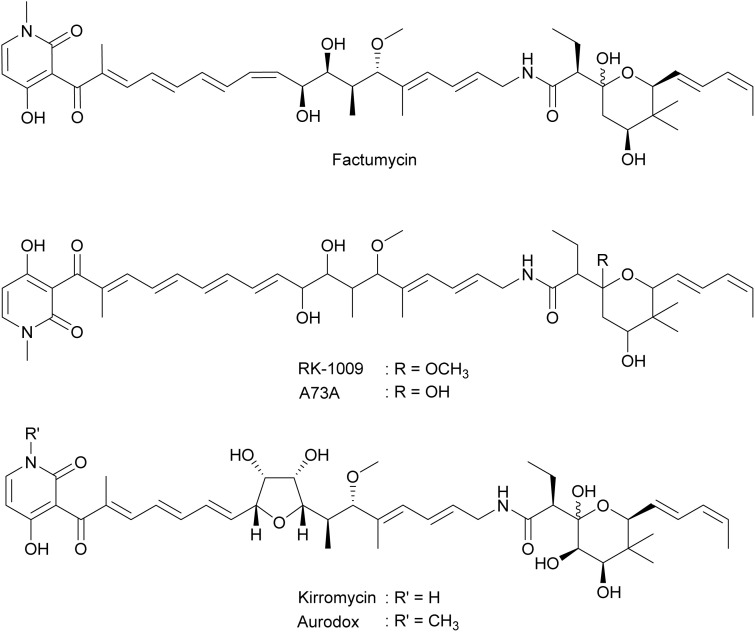
Chemical structures of elfamycins.

Elfamycins (Fig. 19) are a class of structurally diverse antibiotics that specifically target prokaryotic elongation factor thermo unstable (EF-Tu) during protein synthesis.^168,169^ In 1972, the first member of this class, kirromycin, was isolated from *Streptomyces collinus*.^170^ Following the discovery of kirromycin, several of its analogs were also isolated, such as factumycin (A40A) from *Streptomyces lavendulae*,^171^ A73A from *Streptomyces viridifaciens*,^172^ and RK-1009 from *S. griseus.*^173^ These analogs were shown to inhibit plaque formation by bacteriophage B on *S. griseus*,^173^ presumably due to inhibition of phage protein synthesis steps that involve EF-Tu.^174^*N*-Methyl kirromycin (aurodox) in the elfamycin family has been shown to inhibit EF-Tu-assisted tRNA delivery (Fig. 14).^175^ As factumycin, A73A, and RK-1009 share structural similarity with aurodox, they likely interfere with bacterial ribosomes in a similar manner, which eventually inhibits phage protein synthesis and hampers phage reproduction.

### 2.3. Dysregulate protein degradation (acyldepsipeptides)

**Fig. 20:**
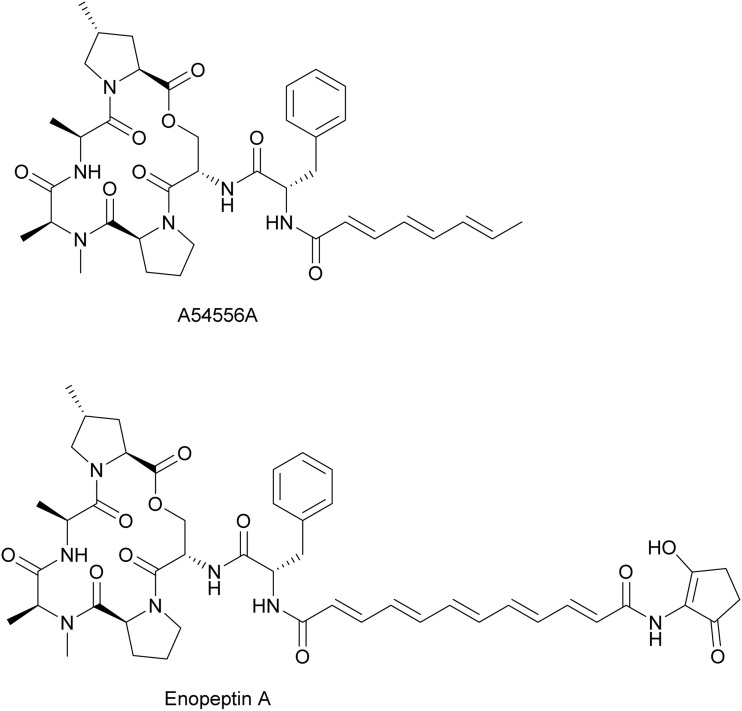
Chemical structures of acyldepsipeptides.

Acyldepsipeptides (Fig. 20) are a relatively new class of antibiotics with promising results against multidrug-resistant pathogens because of their distinct mechanism of action.^176–178^ The first acyldepsipeptide, A54556A, was isolated from *Streptomyces hawaiiensis* in 1985.^179^ Six years later, another member in this class, enopeptin A, was isolated in a screen for anti-phage natural products.^180^ It was shown that enopeptin A produced by *Streptomyces* sp. RK-1051 inhibited plaque formation from bacteriophage B on *S. griseus*.^180^

It is still unclear how enopeptin A inhibits phage proliferation, but it is likely due to the dysregulation of host proteolytic systems. Regulated proteolysis maintains a healthy proteome by identifying and degrading damaged and unneeded proteins.^181^ The caseinolytic protease (Clp) complex is one of the main proteolytic systems in bacteria.^182^ In the Clp complex, 14 units of protease ClpP form a proteolytic chamber, whose activity is regulated by ATPase ClpX/A/C, which recognizes damaged proteins, unfolds them, and threads them into the proteolytic chamber.^183^ Acyldepsipeptides bind at the ATPase pocket on the ClpP surface and dysregulate the proteolytic activity of ClpP complex.^184^ Dysregulation of the host proteolytic system by acyldepsipeptides could be detrimental to the phage infection process in two ways. First, the uncontrolled degradation of nascent peptides could prevent the production of phage proteins. Additionally, some phages encode proteins that regulate host proteolytic systems like the Clp complex. By regulating proteolysis, phages can optimize amino acid flux for their own development.^185^ Therefore, inhibition of Clp may also inhibit phage proliferation by preventing this phage-based reprogramming of proteolysis.

### 2.4. Sequester iron (siderophores)

Siderophores are structurally diverse microbial secondary metabolites with high affinity to iron.^186^ These metabolites are synthesized and secreted by microbes to harvest iron from the environment, and then the iron-bound siderophores are transported back into the cells by specific transporters on the membrane.^186^ Microbial species compete with each other for scarce environmental iron by making structurally distinct siderophores.^187^ Due to the specificity of siderophore transporters, a siderophore made by one species often cannot be utilized by another species, thus sequestering iron away from competitors.^187^

**Fig. 21:**
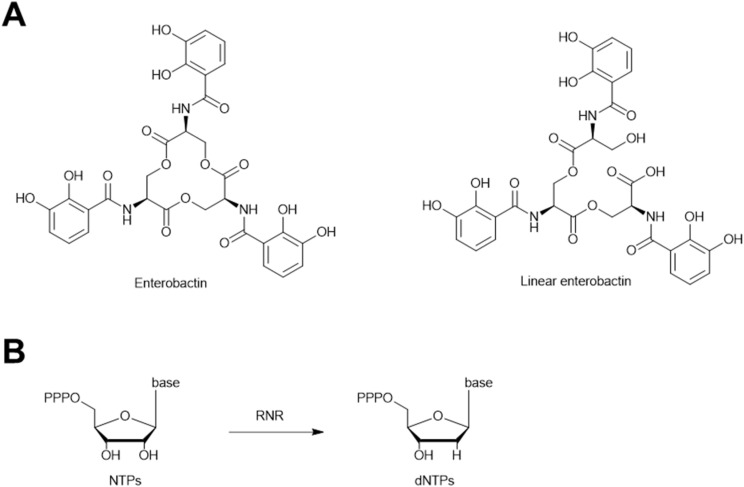
Siderophores inhibit phage infections. (A) Chemical structures of anti-phage siderophores. (B) The reaction catalyzed by ribonucleotide reductase (RNR).

Recently, it has been reported that the *E. coli* siderophores enterobactin and linear enterobactin (Fig. 21A) can repress ICP1 phage proliferation in *Vibrio cholerae* by iron sequestration.^188^ Enterobactin has a very narrow effective range, as it causes a complete growth arrest of *V. cholerae* at concentrations higher than 4 μM. In contrast, linear enterobactin is effective against phages over a wider range of concentrations, because it does not strongly inhibit *V. cholerae* growth even at 200 μM. This special trait of linear enterobactin is likely because *V. cholerae* can pirate linear enterobactin but not enterobactin for its iron uptake.^189^ Therefore, linear enterobactin probably induces a slight iron starvation in *V. cholerae* without completely arresting its growth. This modest iron deficiency in the host appears to inhibit active phage reproduction by delaying phage-mediated cell lysis and reducing the number of new phages produced by each infected cell.^188^

The exact mechanism by which iron deficiency hampers phage proliferation is unclear. Iron is necessary for many cellular processes, and it is plausible that an iron deficiency inhibits several processes that are more essential for phage replication than for host replication.^188^ One hypothesis is that iron deficiency in the host compromises the activity of phage-encoded ribonucleotide reductases (RNRs), thus repressing phage DNA synthesis. RNR is a vital enzyme for DNA synthesis. It converts nucleotides into deoxynucleotides (Fig. 21B).^190^ Phage-encoded RNRs are important for rapid phage DNA synthesis^191^ and effective reproduction.^192^ ICP1 phage encodes a class Ia RNR and a class III RNR on its genome,^193^ both of which require iron as a cofactor.^190^ It has been shown that intracellular iron deficiency caused by an iron chelator can attenuate RNR activity in human cells.^194^ Therefore, it is possible that enterobactin and linear enterobactin sequester iron away from ICP1-infected *V. cholerae*, which inhibits ICP1-encoded RNR activity and impedes rapid ICP1 proliferation. Further experiments are required to distinguish this mechanism from the numerous other influences of iron starvation.

### 2.5. Modify or down-regulate phage receptors

**Fig. 22:**
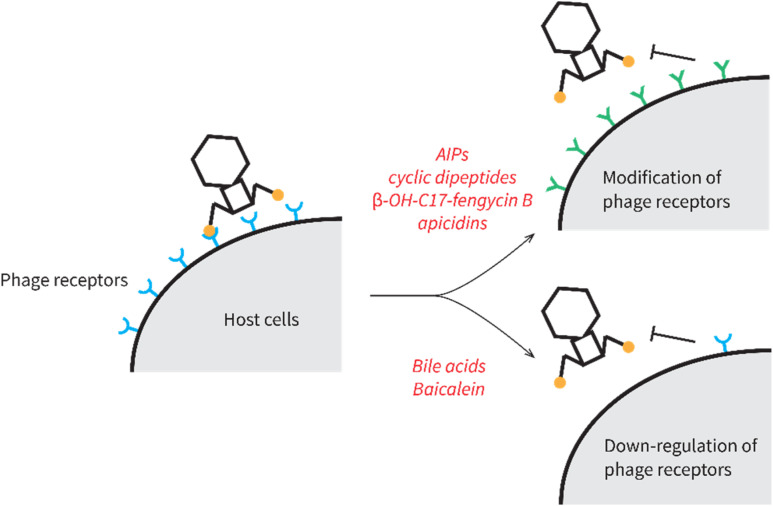
Mechanisms of action of natural products that influence phage adsorption.

In contrast to the previously discussed anti-phage natural products that interfere with phage reproduction within the host cell, others inhibit the initial adsorption of phages to their host surfaces (Fig. 22). Reduced adsorption is mediated by modifications to the bacteria cell surface receptors that phages recognize for binding and infection. These receptors can be modified either qualitatively by changing their composition or quantitatively by decreasing their expression level.

#### 2.5.1. Autoinducing cyclic peptides (AIPs)

**Fig. 23:**
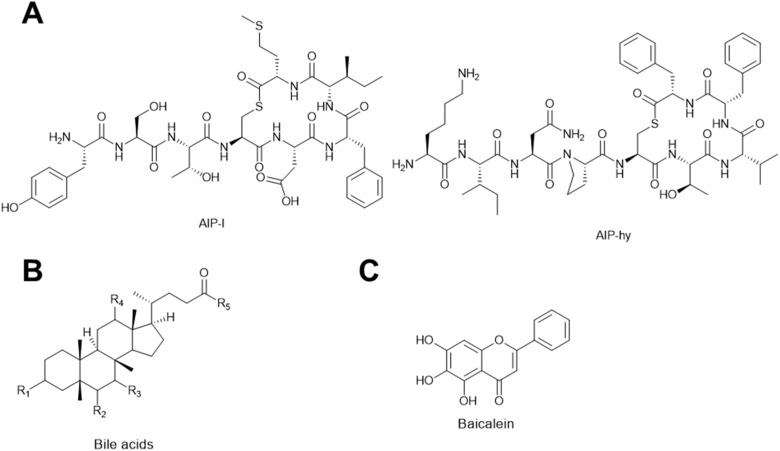
Natural products that modulate or down-regulate phage receptors on the host cell surface. (A) Chemical structures of *agr* inhibitors. (B) Generic chemical structure of bile acids. (C) Chemical structure of baicalein.

Autoinducing cyclic peptides (AIPs) are chemical signals produced by *Staphylococcus* bacteria that encode the *agr* quorum sensing (QS) system (Fig. 23A).^195^ The *agr* QS system coordinates group behaviors of *Staphylococcus* in response to various AIP concentrations at different cell densities.^196,197^ There are four variants (I–IV) of the *agr* system in *S. aureus*, and each variant of the agr system is only induced by its cognate AIP. In fact, non-cognate AIPs produced by other bacteria often inhibit the natural functioning of an *agr* system.^197^ Recently, it was shown that cognate AIP-I can promote Stab20 phage infections in *S. aureus* encoding *agr*-I, while the non-cognate AIP-hy produced by *Staphylococcus hyicus* inhibits phage infections in *S. aureus* encoding *agr*-I, as measured by plaque formation and phage-induced host cell lysis.^198^ The AIPs were shown to influence phage infectivity by modifying the phage receptor on the cell surface. Namely, the AIPs changed the expression levels of *tarM*. TarM is an enzyme that adds α-*N*-acetylglucosamine to the wall teichoic acid (WTA),^199^ which blocks Stab20 phage adsorption.^198^ The cognate AIP-I induces *agr*-I, which represses *tarM* expression, thus facilitating phage adsorption. On the contrary, noncognate AIP-hy inhibits *agr*-I activation, thereby derepressing *tarM* and inhibiting phage adsorption. Furthermore, by co-culture assays, the authors discovered that other *Staphylococcus* strains that frequently co-occur with *S. aureus* on the skin of humans and animals also exhibit an anti-phage effect on *S. aureus*, presumably through secretion of inhibitory non-cognate AIPs.^198^ Therefore, cross-species metabolic interactions can dramatically impact phage infection outcomes in *Staphylococcus*.

#### 2.5.2. Bile acids

Bile acids (Fig. 23B) are a group of cholesterol metabolites with important functions in vertebrate animals, such as facilitating lipid absorption and excretion in the gut, lipid and glucose homeostasis, and immune signaling.^200^ Bile acids are synthesized from cholesterol in the liver as primary bile acids, which are further metabolized by the gut microbiota into secondary bile acids. It was recently discovered that *V. cholerae*, a gut pathogen, became resistant to ICP1 phage infections when exposed to host bile acids.^201^ The authors showed that the phage resistance occurred due to a transient modification of phage receptors on the cell wall in response to a combination of bile acids, anaerobicity, and low pH.^201^ The O-antigen (or outer core polysaccharide) of lipopolysaccharide on the *V. cholerae* cell wall is the receptor of ICP1 phages.^202^ When exposed to bile acids at a low pH under anaerobic conditions, O-antigen synthesis in *V. cholerae* was impaired due to a decrease in O-antigen biosynthetic enzyme levels and a depletion of central carbon metabolites required for constructing O-antigen.^201^ As a result, the decreased O-antigen on the cell surfaces inhibited ICP1 adsorption, thus limiting its infection. This effect may synergize with the aforementioned influence of iron sequestration^188^ to explain transient phage-resistance observed in a prophylaxis phage therapy experiment against *V. cholerae* conducted in animal guts.^203^

#### 2.5.3. Baicalein

Baicalein is a flavonoid compound originally isolated from the roots of *Scutellaria baicalensis* (Fig. 23C).^204^ Recently, it has been shown to inhibit DMS3vir phage infection in *Pseudomonas aeruginosa* through phage adsorption inhibition.^205^ DMS3vir phage requires the type IV pilus of *P. aeruginosa* as its receptor,^206,207^ which is regulated by quorum sensing systems.^208^ The authors proposed that the inhibited phage adsorption was due to the down-regulation of type IV pilus through the inhibition of quorum sensing caused by baicalein.^205^

### 2.6. Activating anti-phage defense systems

**Fig. 24:**
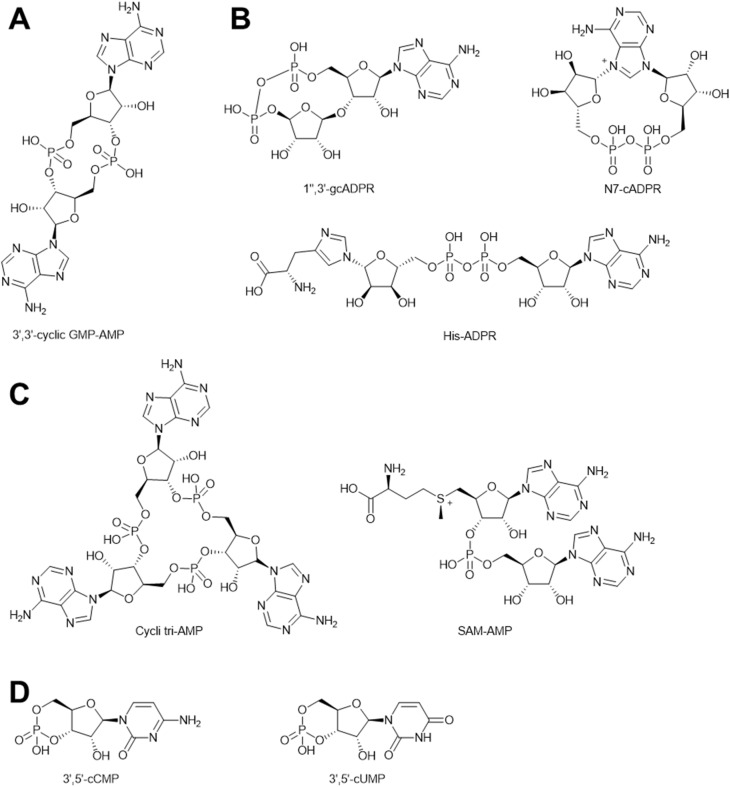
Natural products that activate anti-phage immune systems (A) chemical structure of a representative CBASS signal. (B) Chemical structures of Thoeris signals. (C) Chemical structure of representative type III CRISPR signals. (D) Chemical structures of Pycsar signals.

Over millennia of co-evolution between bacteria and phages, bacteria have acquired hundreds of anti-phage defense systems to provide protection against phage infection.^209^ Most of these systems were cryptic genes within bacterial genomes for decades until recent advances in bioinformatic analysis revealed the mystery of these prokaryotic “immune systems”. A large fraction of these systems rely on nucleotide-derived signaling molecules to abort phage infections,^210^ such as CBASS,^211,212^ Thoeris,^213–215^ type III CRISPR,^216–218^ and Pycsar.^219^ Cumulatively, systems of this type are present in ∼36% of sequenced bacterial genomes.^210^ Generally, these defense systems utilize a sensor protein to sense phage infection and convert cellular nucleotides into secondary signaling molecules. These “immune signals” then bind and activate downstream effector proteins to abort phage infections. CBASS has the most diverse signal molecules among the immune signaling systems, with more than 10 distinct nucleotide signals identified so far. CBASS signal molecules feature cyclic di- or trinucleotide species, with combinations of both purine and pyrimidine bases that are linked through 3′–5′ and/or 2′–5′ phosphodiester bonds (Fig. 24A). Thoeris systems have three types of signals identified so far, which are all derived from cellular NAD^+^ (Fig. 24B). In type III CRISPR systems, two types of signals have been discovered, including cyclic oligoadenylate and SAM-AMP (Fig. 24C). Pycsar systems exclusively synthesize cyclic pyrimidine mononucleotides as signal molecules, such as 3′,5′-cyclic cytosine monophosphate (cCMP) and 3′,5′-cyclic uridine monophosphate (cUMP) (Fig. 24D).

The immune signaling molecules are unique and distinct from other anti-phage natural products in multiple ways. First, although immune signals are specialized to antagonize phage activity, they mostly activate toxic effectors that lead to cell death before phage infection completes.^210^ In contrast, many anti-phage natural products exhibit weak or no toxicity to the host cell at concentrations that inhibit phage infections. Second, the anti-phage action of immune signals has only been demonstrated in a cell-autonomous way. In other words, the signal from an infected cell does not activate defenses in neighboring cells. It would be interesting to investigate if immune signals can also act in non-cell-autonomous way and activate the anti-phage systems in the whole bacterial community to confer “herd immunity” and to shape microbial ecology. Third, the diverse antiphage immune signaling systems provide an opportunity for systematic discovery of anti-phage molecules, since the signals produced by many of the systems remain unknown.

## 3. Phage-promoting natural products

Natural products that promote phage infections have been reported less than those that inhibit phages. However, a revived interest in phage therapy has motivated the discovery of natural products (especially antibiotics^16–19^) that can synergize with phages for antibacterial therapy. Beyond their therapeutic potential, phage-promoting natural products might also play an important role in mediating microbial competition in nature. For example, phage-promoting metabolites produced by one microbe could sensitize its competitors to phage predation, thus giving the producer a competitive advantage over other bacteria.^220^

**Fig. 25:**
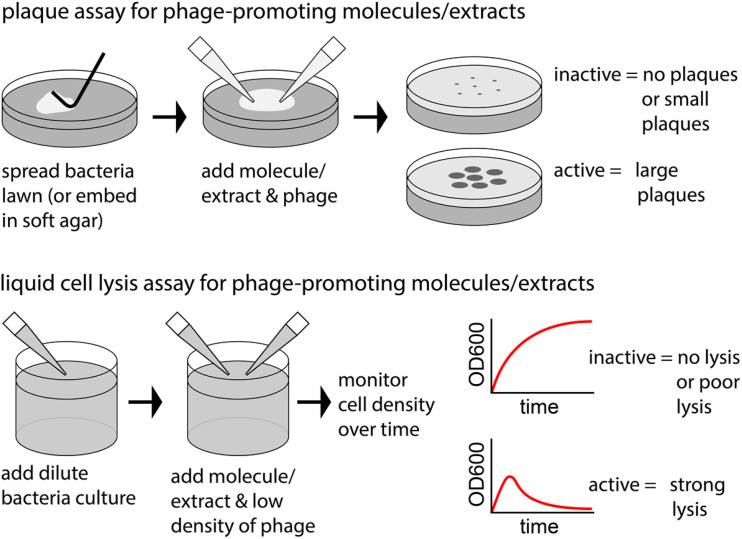
Example experiments to observe phage-promoting natural products.

The phage-promoting activity of natural products can be assessed experimentally in multiple ways. We highlight two methods (Fig. 25) that can reveal selective phage lysis promotion by molecules that are not antimicrobial (at least at the applied dose). One case monitors the increase in area of plaques (areas of phage-induced bacterial lysis on an agar surface). The other case monitors improved phage-induced lysis in liquid culture.

The current known natural products that promote phage infections are discussed in the following sections according to their specific mechanisms.

### 3.1. Inhibit peptidoglycan synthesis (beta-lactams)

**Fig. 26:**
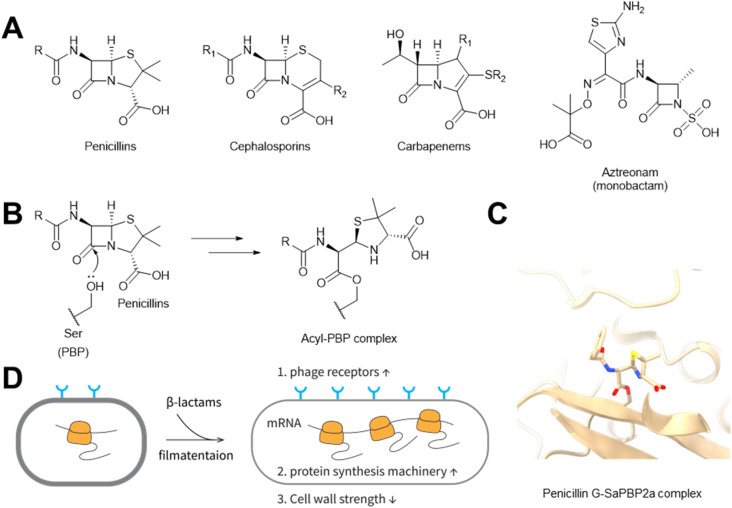
β-Lactams promote phage infection by inhibiting peptidoglycan synthesis. (A) Chemical structures of β-lactams. (B) Formation of an acyl–enzyme complex between β-lactams and PBPs. (C) Structure of penicillin G in complex with PBP2a from *S. aureus*. [PDB: 1MWT]. (D) Possible mechanisms by which filamented cells promote rapid phage proliferation.

β-Lactam compounds are arguably the most widely prescribed antibiotics, representing more than half of all commercially available antibiotics in use.^221^ This family of antibiotics is named after their shared feature: a β-lactam ring (Fig. 26A). In 1929, penicillin was isolated by Alexander Fleming^222^ from *Penicillium rubens*.^223^ In 1947, penicillin was first reported to accelerate phage-mediated host cell lysis in *Staphylococcus aureus.*^224^ Many other classes of β-lactam antibiotics (Fig. 26A) have been isolated from microbes and further expanded by medicinal chemistry efforts,^225^ such as cephalosporins,^226^ carbapenems,^227^ and monobactams.^228^ All of these β-lactam antibiotics synergize with phages to kill a variety of bacterial hosts.^16,229^

The synergy between β-lactam antibiotics and phages is presumably due to the impeded cell wall synthesis caused by β-lactams.^16,230^ One of the key structures of bacterial cell walls is peptidoglycan, whose synthesis is catalyzed by penicillin-binding proteins (PBPs).^231,232^ β-Lactams can occupy the active site of PBPs and form covalent acyl–enzyme complexes that arrest PBP-catalyzed peptidoglycan cross-linking (Fig. 26B and C).^233–235^ At sublethal concentrations of β-lactams, the hampered peptidoglycan synthesis leads to poor cell division and filament formation (Fig. 26D).^16,236^ On one hand, the filamented bacterial cells possess a larger cell surface, which facilitates the phage adsorption step.^230^ On the other hand, the inhibited cell division might cause each bacterial filament “cell” to have more protein synthesis machinery, leading to a larger burst size (the number of new phages produced by each infected cell).^16,230^ Lastly, interrupted peptidoglycan synthesis may also ease the effort of peptidoglycan degradation by endolysins in the phage-mediated cell lysis step, which would expedite cell lysis.^16^

### 3.2. Inhibit stationary phase transition

**Fig. 27:**
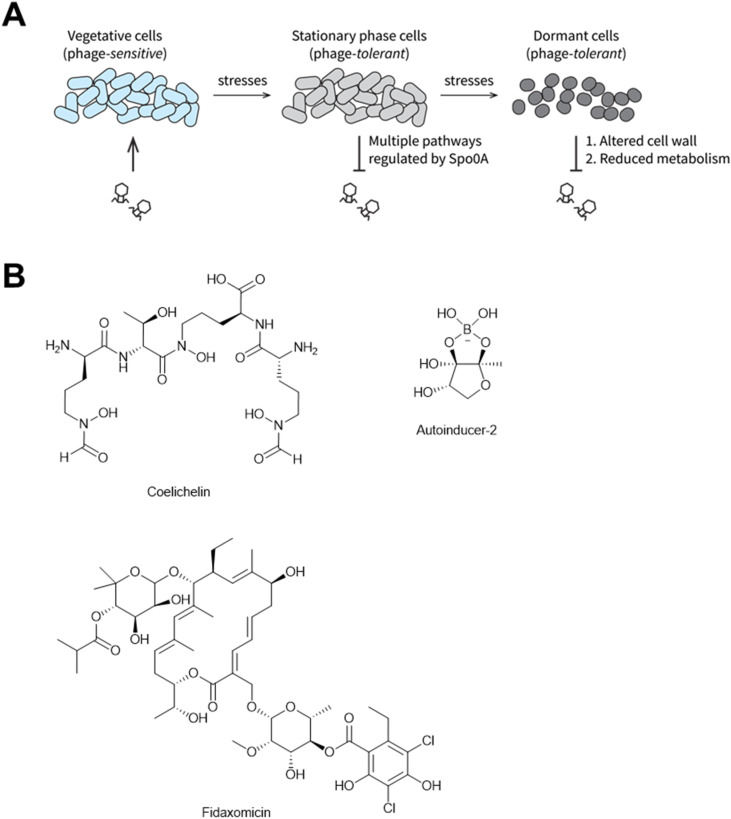
Natural products that promote phage infections by inhibiting stationary phase transition. (A) Mechanisms by which stationary phase transition and dormancy inhibit phage infection. (B) Chemical structures of Spo0A inhibitors.

Transitioning into stationary phase and ultimately cell dormancy are common strategies for bacteria to adapt to environmental stresses.^237^ This transition can further afford recalcitrance to phage infection. For example, in *Bacillus*, multiple pathways regulated by Spo0A during stationary phase transition can repress phage activities (Fig. 27A).^220^ In the dormant state, the altered cell wall^238^ and heavily reduced metabolic activity^237^ can block phage adsorption^238^ and inhibit rapid phage proliferation,^239,240^ respectively (Fig. 27A). Therefore, molecules that inhibit the stationary phase transition and cell dormancy could keep bacterial hosts in their phage-sensitive states, thus promoting phage reproduction. Three examples of natural products with this ability follow.

#### 3.2.1. Siderophores

A recent study showed that a siderophore produced by *Streptomyces*, coelichelin (Fig. 27B), can inhibit the stationary phase transition in *B. subtilis*, thus promoting phage predation on *B. subtilis*.^220^ Iron sequestration caused by coelichelin can block the activation of Spo0A,^220^ the master transcriptional regulator in *B. subtilis* that controls the transition to stationary phase.^241,242^ The authors further showed that coelichelin production gave *Streptomyces* a competitive advantage over *B. subtilis* by sensitizing *B. subtilis* to phage infection.^220^ They found that other siderophores (and even non-siderophore metabolites) also exhibited similar phage-promoting activities.^220^

#### 3.2.2. Other Spo0A inhibitors

Non-siderophore bacterial secondary metabolites have also been shown to inhibit Spo0A activation or expression. Namely, autoinducer-2 (ref. 243) and fidaxomicin (Fig. 27B)^244^ inhibit Spo0A activation in *Bacillus velezensis* and *Clostridioides difficile*, respectively. These Spo0A inhibitors have not been evaluated for their interaction with phages, but hypothetically they could promote phage infection.

Spo0A-regulated dormancy behaviors are found in many bacteria in the Bacillota (Firmicutes) phylum.^245^ The discoveries above suggest that inhibiting the Spo0A-mediated stationary phase transition and sensitizing competitors to phage predation could be a common competition strategy among microbes. It is likely that other natural Spo0A inhibitors exist and remain to be discovered.

### 3.3. Inhibit anti-phage defense systems

As discussed in the previous section, anti-phage immune signaling systems encode protein components that generate or bind small molecule signals.^210^ These components possess cavities for small molecule binding, which could be targets for inhibition or activation by exogenous natural products.

**Fig. 28:**
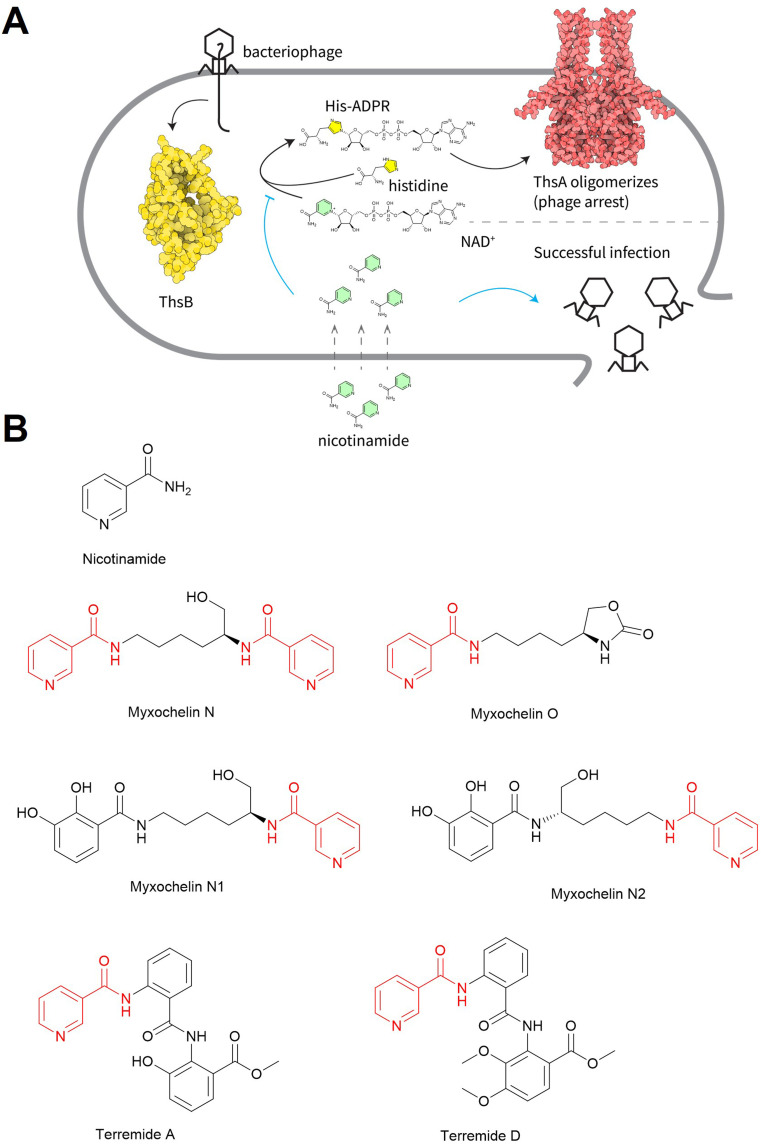
Natural products promote phage infections by inhibiting the type II Thoeris anti-phage system. (A) Mechanism of action of the type II Thoeris system, which can be inhibited by exogenous nicotinamide. (B) Chemical structures of nicotinamide and nicotinamide-containing natural products.

Recently, it was reported that nicotinamide can inhibit the type II Thoeris antiphage system encoded in a wide range of hosts, including *Bacillus amyloliquefaciens*, *P. aeruginosa*, and *Enterococcus faecalis*.^246^ In doing so, it promoted phage predation on these hosts. The type II Thoeris system relies on two proteins, ThsA and ThsB (Fig. 28A).^214^ The ThsB protein can sense phage infection and generate a small molecule alarm signal, histidine-ADP-ribose (His-ADPR). The His-ADPR signal then activates ThsA, which arrests phage replication. Since the first step of His-ADPR biosynthesis is NAD^+^ hydrolysis into nicotinamide and ADPR by the TIR domain of ThsB, excess nicotinamide (Fig. 28A) inhibits NAD^+^ hydrolysis. Therefore, high concentrations of exogenous nicotinamide blocked His-ADPR production and restored phage infectivity.^246^ Beyond nicotinamide, some microbes also produce nicotinamide-containing secondary metabolites, such as myxochelins (Fig. 28B)^247,248^ and terremides (Fig. 28B),^249,250^ which may also inhibit the type II Thoeris system through a similar mechanism of action. Although yet to be demonstrated, nicotinamide and its analogs may also inhibit other immune systems that contain TIR domains.

This discovery is the first demonstration that anti-phage systems can be inhibited by small molecule natural products. Considering the presence of dozens of anti-phage systems, we anticipate that natural products targeting other systems exist and remain to be discovered.

### 3.4. Unknown mechanism (rebaudioside A)

**Fig. 29:**
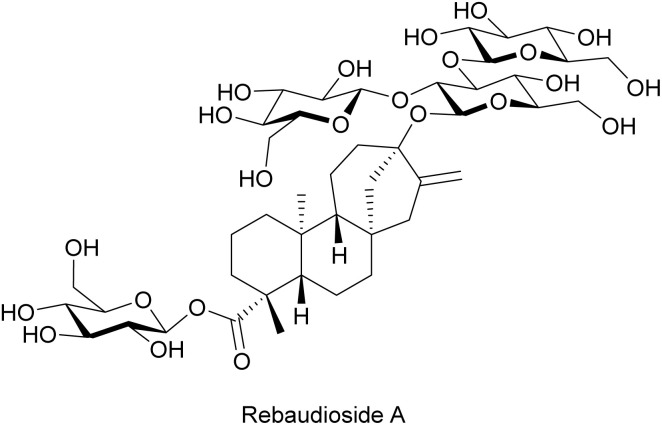
Chemical structures of rebaudioside A.

Rebaudioside A is a natural high-potency sweetener isolated from stevia leaves (Fig. 29).^251^ Recently, it was found that rebaudioside A facilitated phage infection on *Yersinia enterocolitica*.^252^ Preliminary data suggested that phage adsorption was promoted by rebaudioside A treatment.^252^ The increased adsorption might be due to a stabilizing effect imposed on the free phage particles by rebaudioside A.^252^ The phage particles might aggregate in suspensions. Rebaudioside A may prevent phage aggregation, which increases the effective phage titer.^252^ The validation of this hypothesis and the exact mechanism of action of rebaudioside A still require further investigation in the future.

## 4. Lysis/lysogeny-regulating natural products

**Fig. 30:**
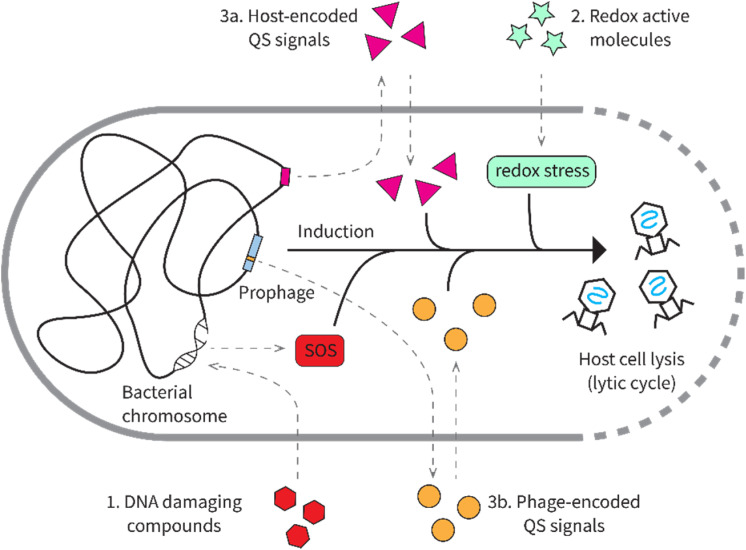
The four major mechanisms of prophage induction.

In contrast to lytic phages that exclusively undergo lytic cycles, temperate phages can undergo both the lytic cycle and the lysogenic cycle.^253,254^ In the lysogenic life cycle, temperate phages integrate their genomes into the chromosome of their host bacteria. Here they lay dormant as “prophages”, replicating along with the host genome and propagating into all of the progeny of that host cell.^253,254^ Temperate phages can then switch back to their lytic life cycle when conditions would benefit lysis—either in response to environmental signals like microbial metabolites or through phage-encoded quorum sensing systems (Fig. 30). In this section, we will review the known microbial metabolites that regulate lysis–lysogeny “decisions” in temperate phages.

### 4.1. Damage DNA

One mechanism that induces many prophages to enter their lytic cycle is DNA damage in the host (Fig. 30). This behavior provides a clear fitness benefit to the prophage. Host cells undergoing excessive DNA damage will likely fail to replicate, which would arrest prophage propagation. Therefore, the phage should benefit from switching to the lytic cycle, where it could disperse dozens of phage particles to infect healthy cells. Since DNA-damaging agents have been attractive lead molecules for antitumor drugs, many prophage-inducing natural products were actually discovered in screens for antitumor drugs using *E. coli* containing the λ prophage.^13–15^ For λ and many other temperate phages, the lysogenic state is maintained by repressor proteins that prevent the transcription of lytic genes.^255^ DNA damaging natural products trigger the SOS response in the host bacterial cells.^256–258^ The activated SOS pathway in the host typically derepresses the lytic genes through one of two mechanisms: (1) autoproteolysis of the repressor proteins in a RecA-dependent manner^259^ or (2) expression of antirepressor proteins that antagonize repressor proteins.^260^

#### 4.1.1. Mitomycins

**Fig. 31:**
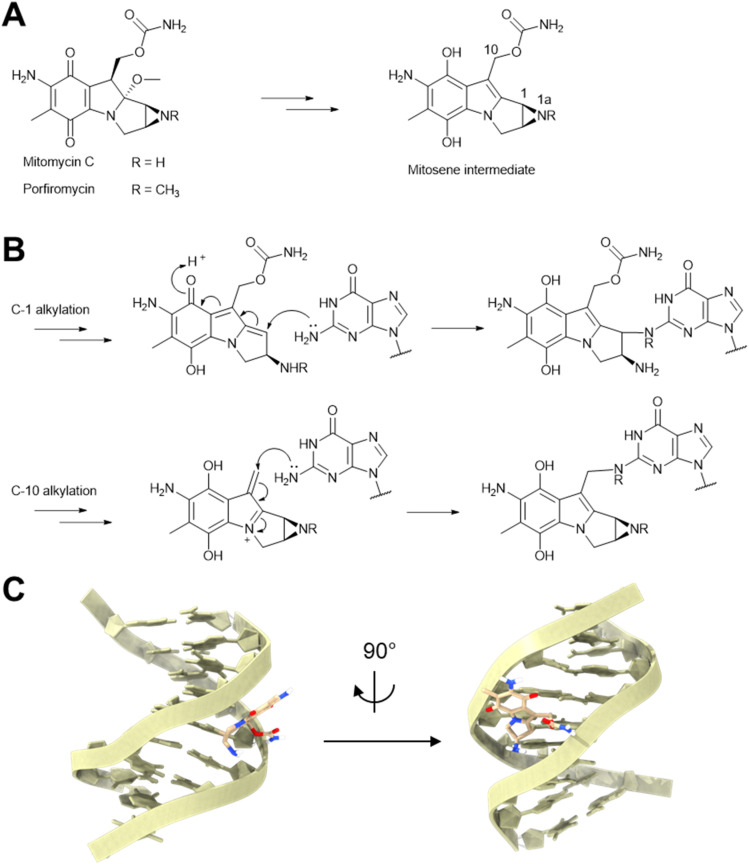
The prophage induction mechanism of mitomycins. (A) Chemical structures of mitomycins and reactive mitosene intermediates. (B) Mechanism of DNA alkylation by mitomycins. (C) Structure of mitomycin C-DNA adduct through C1 alkylation [PDB: 199D].

Mitomycins are a family of potent antibiotics and antitumor drugs, composed of aziridine, quinone, and carbamate moieties arranged on the pyrrolo[1,2-*a*]indole core structure (Fig. 31A).^261^ In 1958, mitomycin C was first isolated from *Streptomyces caespitosus*.^262^ One year later, it was found that mitomycin C could induce the λ prophage in *E. coli* to enter its lytic cycle.^263^ Subsequently, mitomycin C treatment has become a standard protocol for prophage induction. Following the discovery of mitomycin C, the *N*-1a-methyl derivative porfiromycin isolated from *Streptomyces ardus*,^264^ was also shown to induce the lytic cycle of the λ prophage.^14^

The prophage induction capability of mitomycins can be attributed to their ability to alkylate DNA. Following activation through an enzymatic or chemical reduction pathway, mitomycins are converted into reactive mitosene intermediates (Fig. 31A).^265,266^ In the mitosene intermediate, electrophilic centers can be formed at either the C-1 or C-10 position and react with N-2 of guanine (Fig. 31B and C), generating either inter- or intra-strand DNA crosslinks.^267^ The DNA crosslinks activate the SOS response in the host cell,^268^ thus leading to prophage induction.

#### 4.1.2. Azaserine

**Fig. 32:**
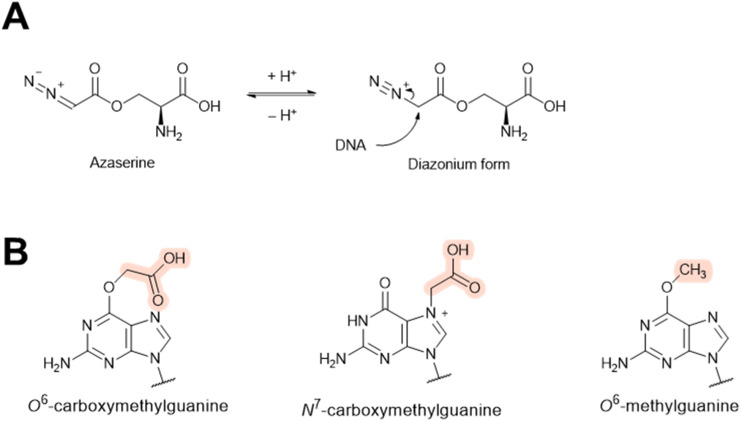
The prophage induction mechanism of azaserine. (A) DNA alkylation mechanism of azaserine. (B) Three possible purine modifications by azaserine (highlighted moiety).

Azaserine is a naturally occurring derivative of serine with an α-diazoester moiety. It exhibits antibiotic and anti-cancer properties (Fig. 32A).^269^ Azaserine was first isolated from *Streptomyces fragilis* in 1954.^270,271^ Shortly following its discovery, azaserine was found to induce λ prophage in *E. coli*.^14,272^ As with mitomycin, the prophage induction activity of azaserine is presumably through a DNA alkylating mechanism. The diazo group in azaserine can undergo protonation to generate the diazonium moiety, which readily decomposes into a carbonium that can alkylate DNA (Fig. 32A).^273,274^ It has been reported that azaserine mainly reacts with purines, and subsequent spontaneous hydrolysis and/or decarboxylation forms *N*^7^-carboxymethylguanine, *O*^6^-carboxymethylguanine, or *O*^6^-methylguanine (Fig. 32B).^275,276^ DNA alkylation by azaserine has been reported to cause extensive DNA damage in bacterial hosts,^277,278^ which subsequently triggers the SOS response.^277,278^ This SOS response likely induces the lytic cycle in a similar manner as above.

#### 4.1.3. Pluramycin A

**Fig. 33:**
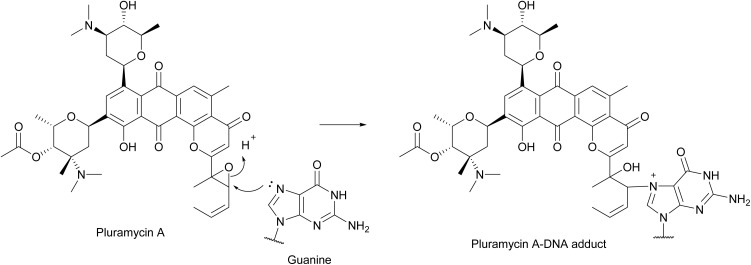
The DNA alkylation mechanism of pluramycin A.

Pluramycin A was first isolated from *Streptomyces pluricolorescens* in 1956 (Fig. 33).^279^ The prophage induction activity of pluramycin A was reported in many studies in the 1960s using λ prophage-containing *E. coli* strains.^13,14,280^ Pluramycin A is structurally similar to the earlier discussed molecule neopluramycin (Fig. 4). Like neopluramycin, pluramycin A is also capable of intercalating DNA.^50^ Notably, the presence of an epoxide ring in pluramycin A (Fig. 33) allows it to react with the N-7 in the guanine base (Fig. 33).^281^ DNA alkylation caused by pluramycin A may induce cellular DNA damage in bacterial hosts^282^ and a subsequent SOS response, therefore inducing prophages to switch to the lytic cycle as discussed above.

#### 4.1.4. Streptozotocin

**Fig. 34:**
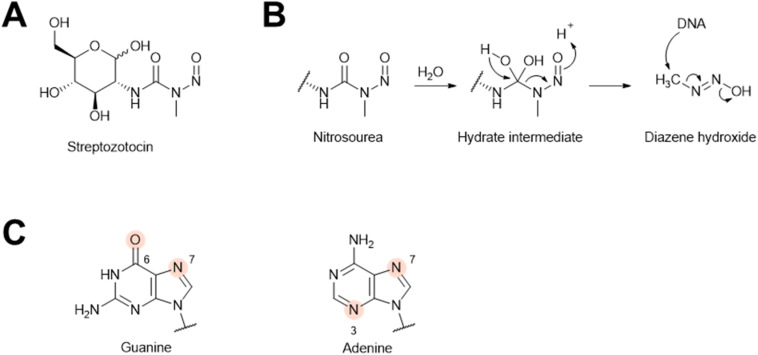
The prophage induction mechanism of streptozotocin. (A) Chemical structure of streptozotocin. (B) DNA alkylation mechanism of the nitrosourea functional group. (C) Possible methylation sites on purines by streptozotocin are highlighted.

Streptozotocin was first isolated from *Streptomyces achromogenes* in 1957 as an antibiotic (Fig. 34A).^283^ Later streptozotocin was shown to induce λ prophage in *E. coli*.^14^ The nitrosourea group in streptozotocin spontaneously decomposes into a diazene hydroxide (Fig. 34B).^284^ Specifically, the nitrosourea first hydrates and then forms diazene hydroxide, which can act as an electrophile for nucleotide bases in DNA (Fig. 34B).^285^ Streptozotocin treatment has been reported to methylate at different sites, such as N-7 and O-6 of guanine and N-3 and N-7 of adenine (Fig. 34C).^286,287^ Due to its DNA-alkylating property, streptozotocin presumably induces prophages *via* the SOS pathway discussed above.

#### 4.1.5. Colibactin

**Fig. 35:**
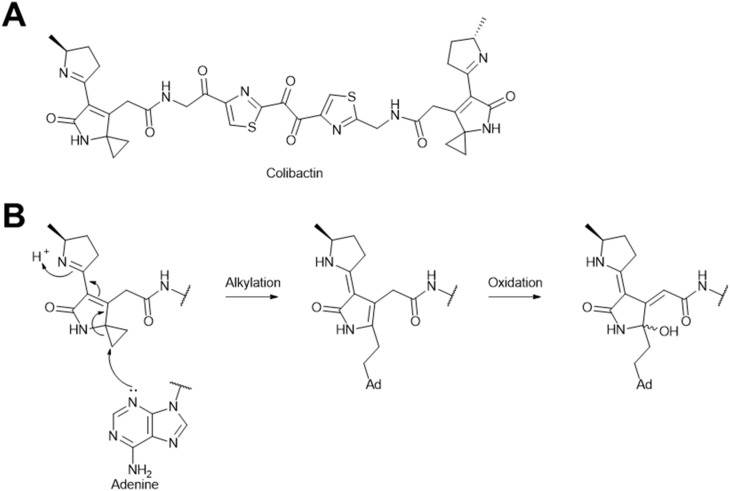
The prophage induction mechanism of colibactin. (A) Chemical structure of colibactin. (B) DNA-alkylation mechanism of colibactin.

Colibactin is a genotoxic metabolite first discovered in 2006, which is synthesized by a 54-kb hybrid nonribosomal peptide synthetase–polyketide synthase (NRPS–PKS) biosynthetic gene cluster (*pks*) in *E. coli*.^288^ Due to its instability and low yield, colibactin has been recalcitrant to isolation, which precluded efforts to solve its chemical structure.^289^ Recently, the structure of colibactin has been resolved through a combinatorial approach of genetics, isotope labeling, tandem mass spectrometry, and chemical synthesis (Fig. 35A).^290,291^ Due to its ability to cause DNA double-stranded breaks,^288^ colibactin has been found to induce the lytic cycle of prophages in a wide range of hosts, such as *pks*^*−*^*E. coli*, *Salmonella enterica*, *S. aureus*, *Citrobacter rodentium*, and *Enterococcus faecium*.^292^ Colibactin possess a pseudodimeric structure with two reactive cyclopropane warheads located at its two ends (Fig. 35A). These warheads specifically alkylate the N-3 of adenine residues (Fig. 35B) and form inter-strand DNA crosslinks.^293^ Since the induction activity is eliminated in a Δ*recA* mutant, the prophage induction by colibactin is believed to occur *via* the RecA-dependent SOS pathway.^292^

#### 4.1.6. Gilvocarcins

In 1982, gilvocarcins V and M (Fig. 5A, isolated from *Streptomyces arenae* 2064) were found to induce prophages in a biochemical prophage induction assay (BIA).^294^ In brief, the bacteria used in this assay harbor an engineered λ prophage that produces β-galactosidase as a reporter of prophage induction conditions.^15^ However, another study published in the same year found that gilvocarcin V did not induce λ prophage^295^ in a standard induction assay.^14^ This discrepancy was clarified later after the discovery that the DNA-alkylating property of gilvocarcin was light dependent (Fig. 6B).^60^ The previous experiments did not control for light as a variable, explaining the inconsistent results. This photo-activated DNA-alkylating activity of gilvocarcin has been shown to cause DNA damage both *in vitro* and in cells.^296–298^ This damage likely triggers the SOS response in host bacteria to induce prophages to enter their lytic cycle through similar mechanisms as discussed above.

#### 4.1.7. Bleomycins

**Fig. 36:**
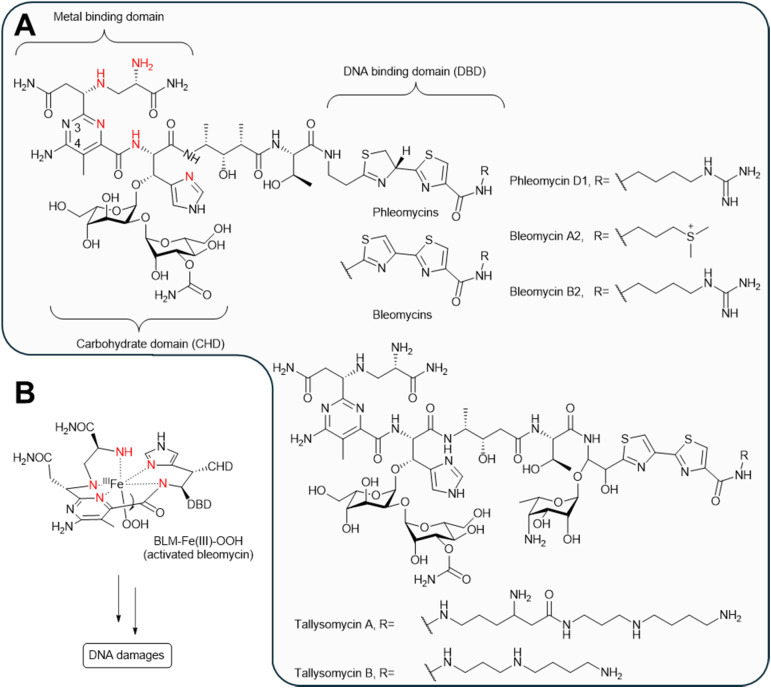
The prophage induction mechanism of bleomycins. (A) Chemical structures of prophage inducing molecules from the bleomycin family. Metal-coordinating residues are colored red. Phleomycin D1 is shown as a representative phleomycin. Bleomycin A2 and B2 are shown as representative bleomycins. (B) Activated bleomycin is key to the DNA degradation activity.

Bleomycins are a family of glycopeptide antibiotics with excellent antitumor activities.^299^ In 1956, phleomycins in this family were first isolated from *Streptomyces*^300^ as a mixture of 12 structurally related components that only differ at the C-terminus of the peptide backbone (Fig. 36A).^301^ Following the discovery of phleomycins, bleomycins were isolated from *Streptomyces verticillus* as a structurally related mixture with A2 and B2 as the major components.^302,303^ Shortly after their discoveries, both phleomycins and bleomycins were reported to induce λ prophage in *E. coli*.^14,304^ Bleomycins also induced PBSH prophage in *B. subtilis*.^304^ Other members in this family, such as tallysomycins A and B (Fig. 36) isolated from *Streptomyces*, also induced λ prophage in *E. coli*.^305^

The prophage induction activity of bleomycins is presumably due to their DNA-damaging mechanisms. The members in the bleomycin family are characterized by a metal-binding domain, a carbohydrate domain, and a DNA-binding domain connected to the former two domains through a linker (Fig. 36A).^306^ The metal-binding domain can complex with redox-active metal ions to form activated bleomycins, which abstract the 4′ hydrogen atom from a deoxyribose residue in DNA, generating DNA strand scission or a 4′-oxidized abasic site (Fig. 36B).^306–308^ These DNA damaging reactions could plausibly trigger the SOS pathway in the bacterial hosts, thus leading to prophage induction through mechanisms discussed earlier.

Beyond prophage induction, bleomycin was also found to inhibit the reproduction of T7 phage on *E. coli*, despite a shorter latent period.^309^ The detailed mechanism of such result is still unclear, but it is possibly related to the DNA degradation caused by bleomycin.

#### 4.1.8. Enediynes

**Fig. 37:**
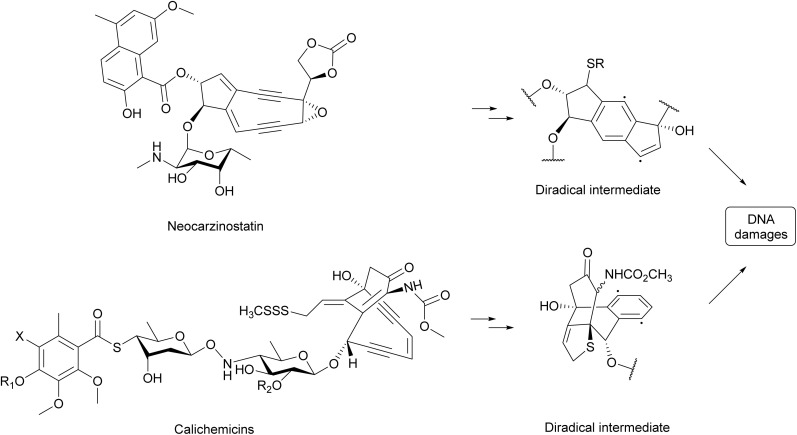
The prophage induction mechanism of enediynes. Chemical structure of prophage inducing enediynes are shown. The diradical intermediates are essential for the DNA damaging activities of enediynes.

Enediyne natural products are anticancer antibiotics with a distinct unsaturated core comprising two acetylenic groups conjugated to a double bond or an incipient double bond.^299,310^ Neocarzinostatin (Fig. 37), the first enediyne antibiotic, was isolated from *Streptomyces carzinostaticus* in 1965 and was reported to induce λ prophage into its lytic cycle.^311^ In a search of novel antitumor agents using the BIA experiment in 1989, calicheamicins (Fig. 37) with prophage induction properties were isolated from *Micromonospora echinosporain*.^312^

Enediynes are known to cause DNA damage through a radical-mediated mechanism.^299,310^ For example, the enediyne structures in both neocarzinostatin^313^ and calicheamicins^310^ can be activated to yield a diradical intermediate (Fig. 37), which abstracts hydrogen atoms from the deoxyribose backbone thus leading to DNA cleavage. DNA damage caused by enediynes likely triggers the SOS response in bacterial hosts, inducing prophages to enter their lytic cycle.

#### 4.1.9. Streptonigrin

**Fig. 38:**
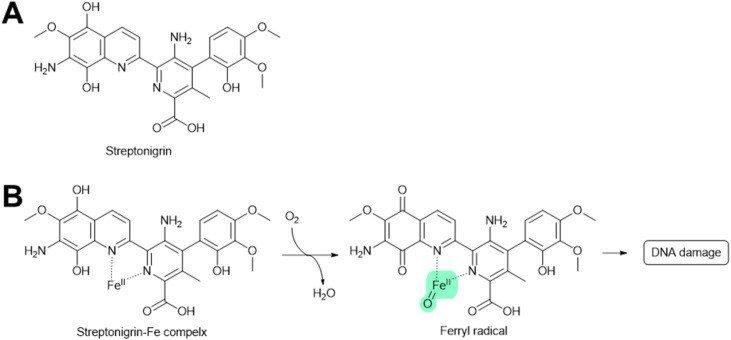
The prophage induction mechanism of streptonigrin. (A) Chemical structure of streptonigrin. (B) Mechanism of formation of DNA-damaging complex from streptonigrin.

Streptonigrin is an aminoquinone antibiotic (Fig. 38A) with antitumor properties that was first isolated from *Streptomyces flocculus* in 1959.^314^ Shortly after its discovery, streptonigrin was reported to induce the lytic cycle in λ and P22 prophages in *E. coli*.^13,315^ The prophage induction activity of streptonigrin is presumably due to its DNA-damaging properties,^315^ which relies on the redox nature of the hydroquinone moiety (Fig. 38B).^316^ The dipyridyl moiety in streptonigrin can complex with Fe^2+^, and under aerobic conditions a ferryl radical can be formed (Fig. 38B).^316^ Due to the DNA binding ability of the strepronigrin–Fe complex, the ferryl radical is in proximity with the DNA, inducing DNA damage (Fig. 38B).^316^ Thus, streptonigrin likely induces prophages through an SOS-mediated pathway following the DNA damage, as discussed for the DNA-alkylating agents in the above section.

#### 4.1.10. Xanthomycin

**Fig. 39:**
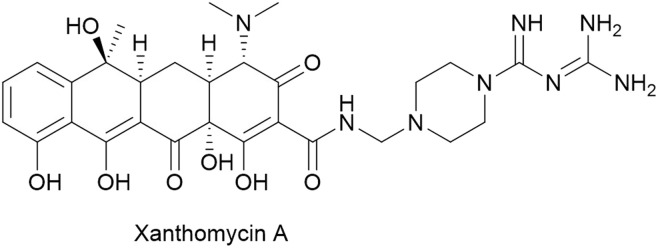
Chemical structure of xanthomycin A.

Xanthomycin belongs to the tetracycline class of antibiotics and was first isolated from *Streptomyces* in 1948,^317^ as a tautomeric mixture of its A and B forms (Fig. 39).^318^ In 1964, it was reported that xanthomycin triggered λ prophage induction.^14^ However, the exact prophage induction mechanism of xanthomycin is still unclear. It was shown that xanthomycin can cause strand scission in PM2 phage DNA *in vitro*, which is presumably due to the free radicals generated by the quinone moiety.^319^ Therefore, xanthomycin might trigger the lytic cycle through DNA damage-associated SOS-dependent pathways as discussed above.

#### 4.1.11. Griseoluteins

**Fig. 40:**
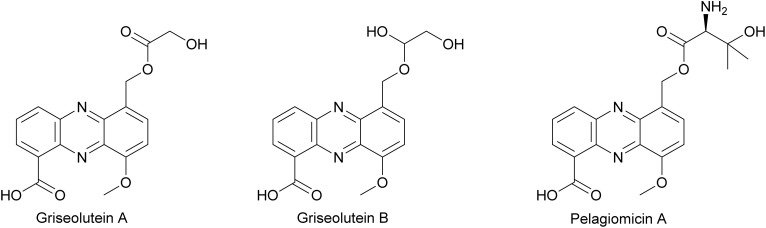
Chemical structures of griseoluteins.

Griseolutein is a phenazine antibiotic that was first isolated from *Streptomyces griseoluteus* in 1950 as a mixture of both A and B forms (Fig. 40).^320^ Shortly after its discovery, griseolutein was found to induce λ prophage in *E. coli*.^14^ In a later study, a structural analog of griseolutein, pelagiomicin A (Fig. 40),^321^ was identified from marine bacteria through the BIA experiment.^322^ This result suggested that griseolutein and pelagiomicin A might induce prophages into their lytic cycles by generating DNA damage, as the BIA assay specifically detects cellular DNA damage.^15^ Both griseolutein and pelagiomicin A feature a phenazine moiety that can cause DNA damage through an iron-dependent pathway.^323^

### 4.2. Induce redox stress (pyocyanin)

**Fig. 41:**
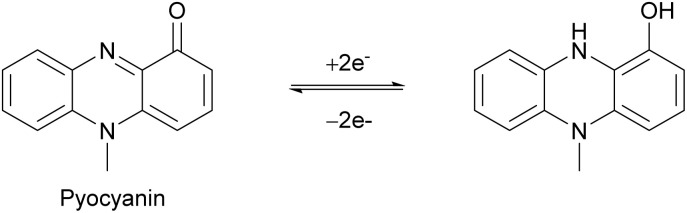
Redox equilibrium of pyocyanin.

Pyocyanin is a common metabolite produced by *P. aeruginosa* (Fig. 41) with a phenazine core structure.^324^ Recently, pyocyanin was shown to induce the lytic cycle in a *S. aureus* prophage.^325^ The authors found that pyocyanin induced the prophage through an SOS-independent mechanism,^325^ in contrast to the DNA-damaging agents discussed above. Under pyocyanin treatment, multiple oxidative stress response genes in *S. aureus* cells were upregulated, indicating that pyocyanin induced a cellular oxidative stress (Fig. 30 and 41).^325^ Notably, the prophage induction by pyocyanin is selective for only certain phages and host strains.^325^ In comparison, oxidative stress caused by hydrogen peroxide promiscuously induces many more phages, presumably through oxidative DNA damage. Pyocyanin-induced oxidative stress in the host cells may induce the lytic cycle through a new mechanism different from the classic de-repression of lytic genes *via* DNA damage.^325^ It is surprising that another class of phenazine-containing molecules, griseoluteins (Fig. 40), was shown to cause prophage induction mainly through the DNA damaging pathway as discussed above. Future work could determine which functional groups and/or cellular conditions dictate the different prophage-inducing mechanisms between pyocyanin and griseoluteins. This discovery also implies that a distinct lytic cycle repression mechanism may be encoded by the pyocyanin-sensitive prophages. They may be uniquely de-repressed in an oxidative cellular environment. Unraveling a novel de-repression mechanism could advance phage biology and open new avenues for the discovery of prophage-inducing molecules. Since most prophage induction experiments have focused on the λ prophage, there may be many other mechanisms and inducers yet to discover.

### 4.3. Regulate quorum sensing

Another mechanism evolved by prophages to determine the optimal time to exit the host cell is to sense the density of nearby host cells. It would only be advantageous for a prophage to leave its host cell if there are plenty of uninfected hosts nearby. Therefore, some prophages have evolved the ability to detect host-encoded quorum sensing (QS) signals. In some cases, prophages even encode their own QS signal (Fig. 30) to assess if nearby hosts have already been lysogenized.

#### 4.3.1. Host-encoded QS signals

**Fig. 42:**
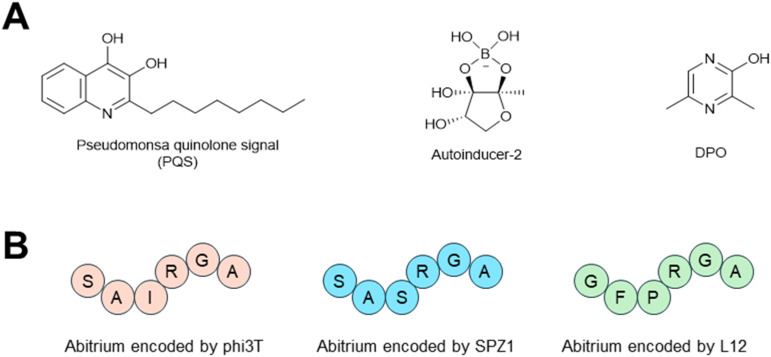
Chemical structures of prophage-regulating quorum sensing (QS) signals. (A) Host-encoded QS signals that can induce the lytic cycle in prophages. (B) Example arbitrium peptides that signal phages to enter and remain in their temperate prophage states.

Group behavior in bacteria is frequently regulated by self-produced QS signal molecules.^326^ Since QS signals accumulate as bacterial density increases, a high level of a QS molecule would signal the presence of a high density of hosts for phage infection. Some prophages have leveraged this correlation of QS signal concentration and host density to regulate entry into their lytic cycles. The first example reported was *Pseudomonas* quinolone signal (PQS, Fig. 42A),^327^ a QS signal produced by *P. aeruginosa*.^328^ PQS was shown to induce prophage entry into its lytic cycle in *Pseudomonas putida*.^327^ However, the molecular mechanism underlying the prophage induction by PQS is still elusive.

A second example was autoinducer-2 (AI-2, Fig. 42A).^329^ This signal was initially identified as a QS signal in *Vibrio harveyi*^330,331^ but was later found to be a widespread QS signal produced by many bacteria.^326^ In 2015, it was found that AI-2 can induce multiple prophages in *E. faecalis* (a bacterium that uses the AI-2 QS signal), although the mechanism of action is still unclear.^329^

A third example is 3,5-dimethylpyrazin-2-ol (DPO, Fig. 42A),^332^ a QS signal in *V. cholerae*.^333^ DPO can induce the *Vibrio parahaemolyticus* VP882 prophage to enter its lytic cycle.^332^ VP882 encodes a DPO-binding QS receptor (VqmA_phage_), which shares homology with the host QS receptor.^332^ Upon DPO binding, VqmA_phage_ induces the expression of an anti-repressor, which was named “quorum-triggered inactivator of cI protein” (Qtip).^332^ Qtip then inactivates the lytic gene repressor, cI, thus triggering the phage lytic program.^332^ The phage particles then disperse to infect the dense population of nearby bacteria.

#### 4.3.2. Phage-encoded QS signals

In addition to hijacking host-encoded QS signals, some phages also encode their own QS signal to coordinate the lysis–lysogeny decision.^334^ This strategy can inform the phage if its nearby host population has already been lysogenized—in which case there is no benefit of trying to “re-infect” those hosts. For example, phages of the SPbeta group encode a six amino-acid-long peptide named “arbitrium” (Fig. 42B), which can regulate their lysis/lysogeny decision.^334^ The arbitrium system consists of three genes: *aimP*, encoding the arbitrium peptide; *aimR*, the arbitrium peptide receptor and transcription factor; and *aimX*, which encodes an AimR-regulated non-coding RNA that represses lysogeny.^334^ Since *aimP* and *aimR* reside in the same operon, AimP and AimR are simultaneously expressed upon phage infection.^334^ AimP is a precursor peptide that is secreted and processed extracellularly into the mature arbitrium peptide. On the other hand, AimR forms a dimer at low phage density, and activates the expression of *aimX*, which represses lysogeny.^334^ Arbitrium peptides can accumulate in the medium and be internalized into the host bacteria by an oligopeptide permease transporter.^334^ A high density of extracellular peptide (indicating nearby lysogenized cells) will lead to an elevated intracellular level of arbitrium peptides, which then bind to AimR and antagonize its activation of *aimX* expression, thus biasing phages to enter (and remain in) the lysogenic cycle.^334^

## 5. Conclusions and outlook

As shown through this manuscript, the long history of natural product research has revealed many metabolites that influence phage behavior. However, the ecological and therapeutic implications of the antagonisms and synergies between natural products and phages are still largely unclear. First, it is worth investigating why the genes encoding these phage-modulating compounds are preserved along the evolutionary path. For example, are some bacterial metabolites that are traditionally thought of as antibiotics actually produced to modulate phage predation as their primary role? Second, with respect to phage-based interventions (*e.g.* phage therapy), an expanded knowledge of the phage-interacting “metabolome” in the actual application settings would help to understand and overcome factors that may diminish phage efficacy. On the other hand, future discoveries of phage-promoting natural products may open new avenues as adjuvants to improve phage efficacy.

Despite many early discoveries of natural products that modulate phage activities, technical limitations and a poor understanding of phage biology obfuscated the molecular mechanisms behind the natural product–phage interactions. In some cases, the mechanisms of action can be speculated from the metabolites' antibiotics or antitumor mechanisms, but generally, elucidation of the phage-influencing mechanisms still requires further investigation.

The recent discovery of natural products inhibiting the Thoeris anti-phage system^246^ suggests that natural inhibitors against many other anti-phage systems may exist. It is possible that microbes have evolved genes to produce such inhibitors to sensitize their neighbors to phages. This behavior would confer a competitive advantage to the producer, as reported in a recent study.^220^ Furthermore, a recent metagenomics study has revealed many biosynthetic gene clusters (BGCs) encoded on phage genomes.^335^ In addition to the proposed functions benefiting the host bacteria,^335^ the natural products encoded by these BGCs might also modulate phage activities, which requires further investigation. Therefore, we believe that nature is filled with phage-produced and phage-influencing natural products—many of which are yet-uncovered or incompletely understood.

## 6. Author contributions

Conceptualization – ZZ, JPG; funding acquisition – JPG; investigation – ZZ, JPG; project administration – JPG; supervision – JPG; visualization – ZZ, JPG; writing (original draft) – ZZ; writing (reviewing & editing) – ZZ, JPG.

## 7. Conflicts of interest

The authors declare no conflicts of interest.

## Data Availability

No primary research results, software or code have been included and no new data were generated or analysed as part of this review.
